# Phase 2b program with sonlicromanol in patients with mitochondrial disease due to m.3243A>G mutation

**DOI:** 10.1093/brain/awae277

**Published:** 2024-11-06

**Authors:** Jan Smeitink, Just van Es, Brigitte Bosman, Mirian C H Janssen, Thomas Klopstock, Grainne Gorman, John Vissing, Gerrit Ruiterkamp, Chris J Edgar, Evertine J Abbink, Rob van Maanen, Oksana Pogoryelova, Claudia Stendel, Almut Bischoff, Ivan Karin, Mahtab Munshi, Anne Kümmel, Lydia Burgert, Christianne Verhaak, Herma Renkema

**Affiliations:** Khondrion B.V., 6534 AT Nijmegen, The Netherlands; Certara Netherlands B.V., 5349 AB Oss, The Netherlands; Khondrion B.V., 6534 AT Nijmegen, The Netherlands; Department of Internal Medicine, Radboud University Medical Center, 6525 GA Nijmegen, The Netherlands; Department of Neurology, Friedrich-Baur-Institute, LMU University Hospital, Ludwig-Maximilians-Universität München, 80336 Munich, Germany; German Center for Neurodegenerative Diseases (DZNE), 81377 Munich, Germany; Munich Cluster for Systems Neurology, 81377 Munich, Germany; Highly Specialised Service for Mitochondrial Disorders of Adults and Children, Newcastle upon Tyne Hospitals NHS Foundation Trust (OP) and Wellcome Centre for Mitochondrial Research, Newcastle University, NE2 4HH Newcastle upon Tyne, UK; Rigshospitalet, University of Copenhagen, DK-2100 Copenhagen, Denmark; Khondrion B.V., 6534 AT Nijmegen, The Netherlands; Cogstate Ltd, SE1 4PG London, UK; Department of Internal Medicine, Radboud University Medical Center, 6525 GA Nijmegen, The Netherlands; Khondrion B.V., 6534 AT Nijmegen, The Netherlands; Highly Specialised Service for Mitochondrial Disorders of Adults and Children, Newcastle upon Tyne Hospitals NHS Foundation Trust (OP) and Wellcome Centre for Mitochondrial Research, Newcastle University, NE2 4HH Newcastle upon Tyne, UK; Department of Neurology, Friedrich-Baur-Institute, LMU University Hospital, Ludwig-Maximilians-Universität München, 80336 Munich, Germany; Department of Neurology, Friedrich-Baur-Institute, LMU University Hospital, Ludwig-Maximilians-Universität München, 80336 Munich, Germany; Department of Neurology, Friedrich-Baur-Institute, LMU University Hospital, Ludwig-Maximilians-Universität München, 80336 Munich, Germany; Certara Netherlands B.V., 5349 AB Oss, The Netherlands; IntiQuan AG, 4051 Basel, Switzerland; IntiQuan AG, 4051 Basel, Switzerland; Department of Psychology, Radboud University Medical Center, 6525 GA Nijmegen, The Netherlands; Khondrion B.V., 6534 AT Nijmegen, The Netherlands

**Keywords:** primary mitochondrial disease, MELAS, MIDD, m.3243A>G, sonlicromanol

## Abstract

Mitochondrial disease incorporates a group of rare conditions with no approved treatment to date, except for Leber hereditary optic neuropathy. Therapeutic options to alleviate the symptoms of mitochondrial disease are urgently needed. Sonlicromanol is a promising candidate, as it positively alters the key metabolic and inflammatory pathways associated with mitochondrial disease. Sonlicromanol is a reductive and oxidative distress modulator, selectively inhibiting microsomal prostaglandin E1 synthase activity. This phase 2b program, aimed at evaluating sonlicromanol in adults with m.3243A>G mutation and primary mitochondrial disease, consisted of a randomized controlled (RCT) study (dose-selection) followed by a 52-week open-label extension study (EXT, long-term tolerability, safety and efficacy of sonlicromanol).

Patients were randomized (1:1:1) to receive 100 or 50 mg sonlicromanol or placebo twice daily (bid) for 28 days with a ≥2-week wash-out period between treatments. Patients who completed the RCT study entered the EXT study, wherein they received 100 mg sonlicromanol bid.

Overall, 27 patients were randomized (24 RCT patients completed all periods). Fifteen patients entered the EXT, and 12 patients were included in the EXT analysis set. All patients reported good tolerability and favourable safety, with pharmacokinetic results comparable to the earlier phase 2a study.

The RCT primary end point [change from placebo in the attentional domain of the cognition score (visual identification; Cogstate IDN)] did not reach statistical significance. Using a categorization of the subject's period baseline a treatment effect over placebo was observed if their baseline was more affected (*P* = 0.0338). Using this approach, there were signals of improvements over placebo in at least one dose in the Beck Depression Inventory (BDI, *P* = 0.0143), Cognitive Failure Questionnaire (*P* = 0.0113) and the depression subscale of the Hospital Anxiety and Depression Scale (*P* = 0.0256).

Statistically and/or clinically meaningful improvements were observed in the patient- and clinician-reported outcome measures at the end of the EXT study [Test of Attentional Performance (TAP) with alarm, *P* = 0.0102; TAP without alarm, *P* = 0.0047; BDI somatic, *P* = 0.0261; BDI total, *P* = 0.0563; SF12 physical component score, *P* = 0.0008]. Seven of nine domains of RAND-Short Form-36-like SF-36 pain improved (*P* = 0.0105). Other promising results were observed in the Neuro-Quality of Life Short Form-Fatigue Scale (*P* = 0.0036), mini-Balance Evaluation Systems test (*P* = 0.0009), McGill Pain Questionnaire (*P* = 0.0105), EuroQol EQ-5D-5L-Visual Analog Scale (*P* = 0.0213) and EQ-5D-5L-Index (*P* = 0.0173). Most patients showed improvement in the Five Times Sit-To-Stand Test.

Sonlicromanol was well-tolerated and demonstrated a favourable benefit/risk ratio for up to 1 year. Sonlicromanol was efficacious in patients when affected at baseline, as seen across a variety of clinically relevant domains. Long-term treatment showed more pronounced changes from baseline.

## Teaser

The m.3243A>G variant of the mitochondrial *MT-TL1* gene is the most common genetic cause of primary mitochondrial disease. Smeitink *et al.* evaluate the pharmacokinetics, safety and efficacy of sonlicromanol, a small molecule that targets pathways involved in oxidative stress and inflammation, in adults with the m.3243A>G variant.

## Introduction

The m.3243A>G variant of the mitochondrial *MT-TL1* gene is the most common genetic defect causing primary mitochondrial disease [prevalence: 3.5 (2.7–4.4) per 100 000 live births].^1-3^ Primary mitochondrial disease associated with the m.3243A>G variant in the mitochondrial genome was originally referred to as MELAS syndrome (mitochondrial encephalopathy, lactic acidosis and stroke-like episodes).^1,4^ However, it includes a spectrum of phenotypes like classic MELAS, MIDD (maternally inherited diabetes mellitus and deafness) syndrome, MP (mixed phenotypes) and CPEO (chronic progressive external ophthalmoplegia).^5,6^ Of these, only 5%–10% of patients have classic MELAS, while >80% have MP or MIDD (with an almost equal prevalence).^6^ The extent of heteroplasmy (the ratio between the mutant and the co-existing wild-type mitochondrial DNA in tissue cells) is one of the factors causing clinical heterogeneity, but other factors are also at play and not yet identified.

The m.3243A>G variant of the *MT-TL1* gene is associated with tRNA^Leu(UUR)^,^1^ and causes a translational defect leading to isolated or combined Complex I oxidative phosphorylation deficiency and subsequent hampered oxidative phosphorylation (OXPHOS).^7^ This defect enhances the production of reactive oxygen species (oxidative distress), alters cellular redox tone (reductive distress) and triggers inflammatory responses.^8,9^

Sonlicromanol is a chromanol piperidine that has been shown to improve cellular redox status in preclinical models. It activates the thioredoxin system/peroxiredoxin enzyme machinery and attenuates induced lipid peroxidation, thereby preventing ferroptosis. Furthermore, it selectively inhibits microsomal prostaglandin synthase E-1-mediated PGE2 biosynthesis and attenuates inflammation.^10,11^ In Complex I-deficient mice, long-term treatment with sonlicromanol is reported to retain the brain's microstructural coherence in the external capsule (involved in cognition) by reducing lipid peroxidation.^12^ Studies have also shown that sonlicromanol significantly improved the performance of rotarod and gait, reduced the degeneration of retinal ganglion cells and improved the lifespan of these mice.^12,13^ Sonlicromanol improved neuronal network function and transcriptome changes in induced pluripotent stem cell (iPSC)-derived human neurons from patients with m.3243A>G mutation.^14^ Previous studies have reported that sonlicromanol has high bioavailability; it crosses the blood–brain barrier and exhibits excellent pharmacokinetic properties. Furthermore, it is well tolerated and has a favourable risk/benefit ratio.^15,16^ Muscle weakness, chronic fatigue, pain and cognitive decline are some of the challenges faced by patients with mitochondrial disease due to m.3243A>G mutation. This may lead to social, emotional and economic impairment of different aspects of daily function in these patients. This observation was also reported by the Voice of the Patient Report of the United Mitochondrial Disease Foundation.^17^ An earlier exploratory phase 2a study conducted in patients with m.3243A>G mutation demonstrated that sonlicromanol has favourable safety, good tolerability and an effect on mood and cognition outcome measures.^15^ In this study, we report the results of a phase 2b program that consisted of a randomized placebo-controlled study (RCT, dose selection) followed by a 52-week open-label extension (EXT).

To extensively study the effect of sonlicromanol on the plethora of symptoms in patients with m.3243A>G mutation,^18^ the RCT was designed to include selected outcome measures evaluated in the earlier phase 2a study and not previously studied other outcome measures.^15^ The change from placebo (CFP) in the visual identification task (IDN, measure of attention) was selected as the primary end point. Long-term safety data were collected during the EXT that also contained additional outcome measures that might change in the slowly disease-progressing m.3243A>G mutation patients.

## Materials and methods

### Study design and participants

The protocols and amendments of the phase 2b program (RCT and EXT) were approved by independent ethics committees and competent authorities in the Netherlands, Germany, the UK and Denmark. The phase 2b program was conducted in accordance with the principles of the Declaration of Helsinki, the International Council for Harmonization of Technical Requirements for Pharmaceuticals for Human Use, Good Clinical Practice guidelines and all the applicable national and local regulatory requirements. Written informed consent was obtained from all the patients participating in this program.

**Figure 1:**
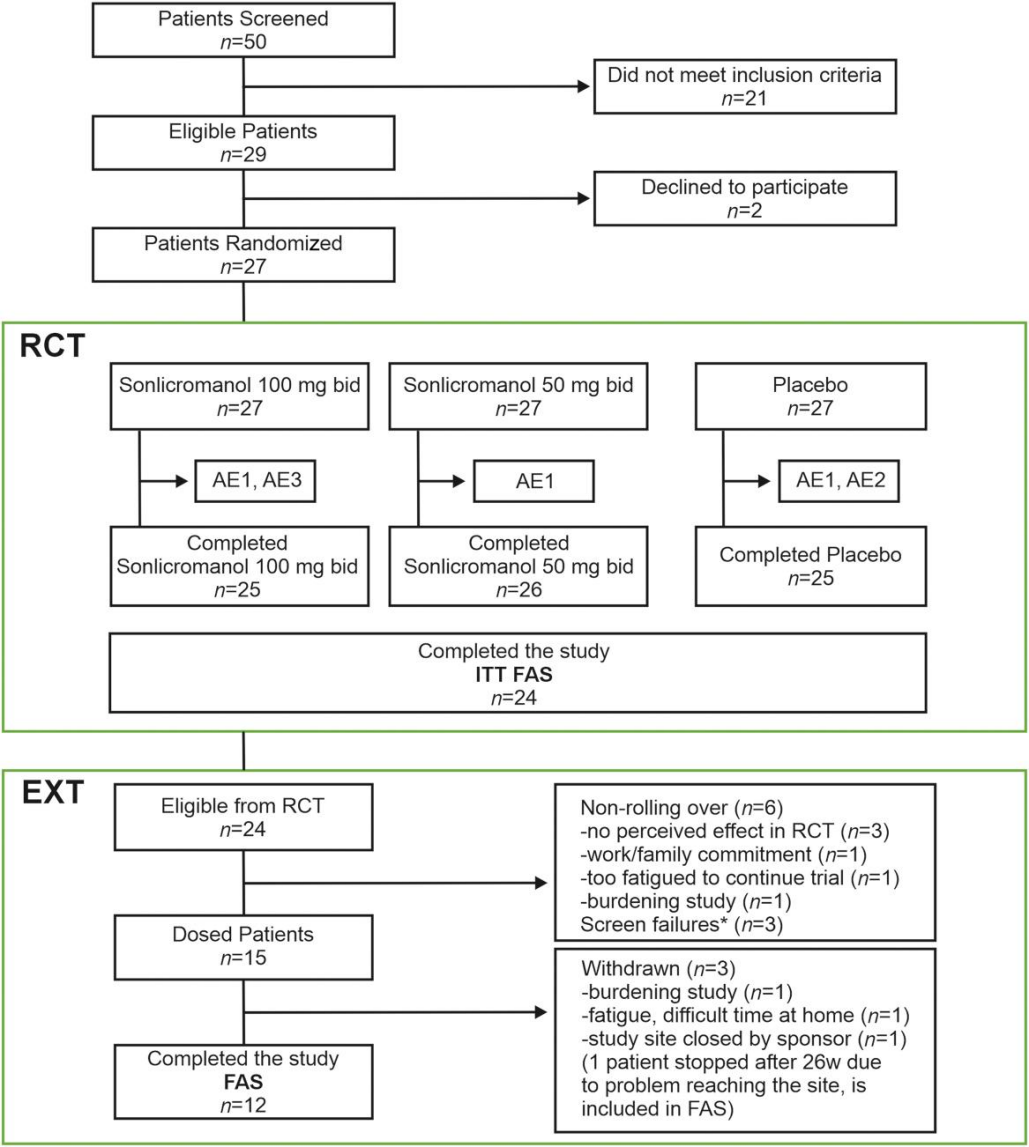
**Phase 2b sonlicromanol program design and patient disposition**. Patients were screened. The eligible and randomized patients were included in the randomized controlled trial (RCT) and the 52-week extension (EXT) study. The intent-to-treat (ITT) population included all patients who were randomized for this program and had a post-baseline assessment of at least one efficacy parameter. The full analysis set (FAS) population consisted of all the patients who received study medication in this program and had at least one on-treatment efficacy assessment after the first drug intake. The safety population included all the patients who received at least one dose of study medication, irrespective of satisfying other criteria. Adverse effect (AE) 1 = medical device site reaction during treatment period (TP) 1; AE2 = ECG T-wave amplitude decrease in TP2; AE3 = ECG changes after coronavirus disease 2019 (COVID-19) vaccination during TP3. Baseline screening before the EXT revealed three screen failures: eGFR 49 ml/min [exclusion criteria (e.c.) 5b], ECG negative T waves (e.c. 6), Fridericia-corrected QT interval (QTcF) 475 ms (e.c. 6). bid = twice daily.

The RCT (NCT04165239; EudraCT Number: 2019-000599-40) was a three-way cross-over, 28-day, double-blind study with a ≥2-week wash-out period between treatments that evaluated the safety and efficacy of the two doses [50 mg twice daily (bid) and 100 mg bid] of sonlicromanol versus placebo in patients with m.3243A>G mutation. Patients who completed the RCT were eligible to enter the EXT (52 weeks; sonlicromanol 100 mg bid, NCT04604548; EudraCT Number: 2020-000832-23) (Fig. 1). For entrance to the EXT arm, patients were again screened against the inclusion and exclusion criteria mainly for safety reasons. Details of the inclusion and exclusion criteria are provided in the Supplementary material, Appendix.

### Randomization and masking

Following the screening, all eligible patients were randomized (1:1:1) on Day 1 of the first treatment period to a treatment sequence starting with either sonlicromanol 50 mg bid, sonlicromanol 100 mg bid or placebo bid. The treatment order was determined using the block randomization technique (three kit numbers were assigned, each representing one treatment regimen). There were no replacements for patients dropping out of the study prior to terminating treatment period 3 (TP3).

### Pharmacokinetic analysis

#### Randomized controlled trial

Six blood samples were collected from each patient on Day 28 of each treatment period; the concentrations of sonlicromanol and its active metabolite KH183 (KH176m) were determined at predose and at 1-, 2-, 4-, 8- and 12-h post-dose. A total of four pharmacokinetic profiles were obtained for each patient: sonlicromanol and KH183 for both doses. The pharmacokinetic parameters evaluated included maximum plasma concentration (C_max_), the area under the curve until the last time with a concentration above the limit of quantification (AUC_last_) and the time to achieve C_max_ (T_max_). The pharmacokinetic parameters for sonlicromanol and KH183 were evaluated using non-compartmental analysis (R 4.1.3 with package IQnca 1.2.0).

#### Open-label extension study

Four blood samples were also collected per patient at Week 52 to determine the concentrations of sonlicromanol and KH183 at predose and 0.5-, 2- and 5-h post-dose. Using a compartmental model-based approach, pharmacokinetic observations in RCT were compared with those in EXT through simulations.

### Efficacy outcome measures

#### Randomized controlled trial

The primary end point of the RCT was the Cogstate IDN (part of the Cogstate computerized cognitive testing battery).^19^ All Cogstate outcomes’ reaction times were transformed to *z*-scores, in which a higher score is better.

Secondary end points were executive functioning, psychomotor function, working memory, visual learning, verbal learning of the Cogstate computerized cognitive testing battery [summarized into the Global Composite Score (GCS)] and the alertness subtest of the Test of Attentional Performance (TAP), with reaction time (in ms) under two conditions (with and without alarm).^20^ The Hospital Anxiety and Depression Scale (HADS) was used to measure the core symptoms of anxiety and depression [seven-item questionnaire subscales HADS-A (for anxiety) and HADS-D (for depression)].^21^ Depression was also measured using the 21-item Beck Depression Inventory (BDI) scale composed of items related to symptoms of depression.^22^

Disease severity was measured with the Newcastle Mitochondrial Disease Adults Scale (NMDAS), which was developed to be analysed annually.^23^ Therefore, the scores obtained in the 28-day RCT were not considered valid. The SF-12 v2, assessed as section IV of the NMDAS, was used as a generic measure of quality of life (QoL). From SF-12, two sub-scores [Physical Component Score (PCS) and Mental Component Score (MCS), both norm-based scores (T-scores)] were derived. The number of days a patient experienced headaches, as well as the intensity and the duration of headache episodes, was evaluated along with the use of medications to relieve headaches; hearing loss was evaluated using pure tone audiometry (PTA); and the function of a patient's olfactory system was measured using the University of Pennsylvania Smell Identification Test (UPSIT).^24^ Fatigue was measured using the self-reported Neuro-QoL Short Form-Fatigue scale (NQF).^25^ The scores were converted to a T-score. The 25-item Cognitive Failure Questionnaire (CFQ) was used to measure subjective cognitive functioning, and the frequency of cognitive errors was assessed.^26^

#### Open-label extension study

In addition to the above efficacy outcome measures, the Five Times Sit-to-Stand Test was used to determine the functional strength and balance of lower limbs.^27^ Balance was measured by performing the mini-Balance Evaluation Systems test (mini-BESTest),^28,29^ which is a comprehensive, unidimensional, brief 14-item balance scale used to measure the important aspects of a dynamic balance control. The short form of the McGill Pain Questionnaire (SF-MPQ) was used to assess the sensory and emotional experiences of pain.^30^ The QoL related to health was evaluated using the 36-item multi-dimension, self-reported RAND Short Form-36 (SF-36) questionnaire^31^ and the EQ-5D-5L^32^ Furthermore, the clinician-scored global impression of change (CGIC), the patient-reported global impression of change (PGIC) and the patient's most bothersome symptom assessment (MBSA) were all scored on a seven-point Likert scale.^33^

Treatment effects were assessed every 13 weeks, allowing extension of the statistical testing. For the NMDAS, Cogstate test battery, BDI, HADS, CFQ and NQF assessment data from the follow-up visit in the RCT were used if this visit occurred within 2 weeks of the predose visit of the EXT.

### Safety and tolerability assessments

Safety assessments included the monitoring of adverse events, safety laboratory tests, vital sign measurements, 12-lead ECGs, thyroid sonography (size and echo density) and physical examination findings. Safety in the EXT was monitored every 3 months by an independent data safety monitoring board.

### Statistical analysis

#### Randomized controlled trial

All efficacy analyses were performed using the intent-to-treat final analysis set (ITT-FAS) (defined as all patients who received study medication and who had at least one on-treatment efficacy assessment after the first drug intake). Safety was evaluated using the safety analysis set (all randomized patients who received at least one dose of study medication were analysed as treated). The pharmacokinetic analysis set included all patients who received the study drug and had at least one measurable drug concentration post dosing.

For clinical outcomes, a superiority analysis was performed using a three-period, three-treatment cross-over model. It compared the CFP at Day 28 for both patient groups (50 mg bid and 100 mg bid). Treatment effects were investigated using a repeated mixed effect model (with treatment and period as fixed effects and patient as the repeated measure). Treatment estimates and 95% confidence intervals (CI) of both active doses were estimated by performing a mixed-model comparison with the placebo.

To identify additional patient characteristics that appeared important in describing the results across multiple end points, more sensitive *post hoc* exploratory analyses were performed. It can be reasoned that it is plausible that the baseline characteristics of the patient at the start of each study period may influence the degree of improvement for that patient after treatment. Therefore, the baseline scores for each treatment period were categorized into one of two severity groups for each end point. This was achieved using normative or -off scores for BDI, CFQ, HADS-A, HADS-D, NQF, SF-12-MCS, SF-12-PCS and NMDAS or the distribution of baseline scores using median values as a cut-off (≤median versus >median; median calculated across all subjects and periods) for the other end points [Cogstate tests, TAP (with/without alarm), NMDAS Section I, II, III; Supplementary Table 1], resulting in a classification of patient periods into dichotomous baseline categories; affected versus normal, more affected versus less affected and severe versus mild/moderate.

A *post hoc* modified mixed model (adjusted model) analysis was then performed in which additional covariates were introduced to the mixed model: the period baseline value categorized into a binary variable (affected versus normal, more affected versus less affected or severe versus mild/moderate, depending on the outcome measure) and the interaction of this categorized period baseline value with the treatment factor.

The change from baseline (CFB) of each treatment group and the differences in CFB between the active treatment groups versus placebo were calculated within each period baseline category.

One extreme outlier in the Cogstate IDN score was excluded from the analysis. This patient had highly fluctuating *z*-scores (between −6.6 and −1.7), suggesting confounding problems while performing this test. The number of patients affected for each outcome measure and the baseline averages for both categories are shown in Supplementary Table 2.

The *post hoc* exploratory analyses were done after database lock and unblinding. No correction for the multitude of outcome measures was performed for any of the analyses beyond the primary end point analysis, according to the statistical analysis plan.

#### Open-label extension study

The mean CFB was analysed using a paired analysis test. A positive response was defined as CFBs in the direction of improvement. Statistical *P*-values were calculated subsequently. The data obtained at the 13-, 26- and 39-week assessments were incorporated by performing a repeated measures analysis of covariance (ANCOVA) model, including visit as a fixed effect and baseline as a covariate) to obtain a treatment estimate with a *P*-value and 95% CI. It should be noted that no correction for the multitude of outcome measures was made for any of the analyses beyond the initial statistical analysis plan, in line with the exploratory nature of the extension study.

For all outcome measures, the cut-off values for the affected range and the values of minimal clinically important difference (MCID) were obtained from the literature if available (Supplementary Table 1) and, if not, an estimate of 0.50 SD was used.^34^ This was based on the concept of MCID ‘clinical meaningfulness’, defined as ‘the smallest difference in score in the domain of interest, which patients perceive as beneficial and which would mandate, in the absence of troublesome side effects and excessive costs, a change in the patient's management'.^35^ While the SD of baseline data can be used as an estimate for MCID if an evaluation study is not available due to particular limitations, such as in a rare disease, a review of various studies suggested that a MCID corresponding to an effect size of 0.30–0.50 SD can be used as a general estimate for different outcome instruments (questionnaires) and study settings (observational cohort studies, RCTs, meta-analyses).^34^

## Results

### Participant characteristics

A total of 50 potentially eligible patients were identified from the mitochondrial disease registries in the Netherlands, Germany (mitoNET registry), UK and Denmark between January 2020 and December 2021. Of these, 27 patients were found eligible to participate in the three-way crossover phase 2b program and randomized to a treatment sequence starting with either sonlicromanol 50 mg bid, sonlicromanol 100 mg bid or placebo bid (Fig. 1). Table 1 and Supplementary Table 3 present the baseline characteristics of the ITT FAS population (*n* = 27). Fifteen of the 27 randomized patients were diagnosed with MIDD (55.5%), 10 (37.0%) had MP, one (3.5%) had classic MELAS and one (3.5%) had CPEO.

**Table 1:** Baseline characteristics of the randomized controlled trial intent-to-treat population (*n* = 27)

Demographic data	(*n* = 27)
Age, years, mean (SD)	46.0 (10.8)
Female patients, *n* (%)	20 (74.1)
Race, white, *n* (%)	27 (100)
BMI, kg/m^2^, mean (SD)	22.55 (3.94)
**Medical history** ^a^	
Migraine, *n* (%)	10 (37)
Hearing loss, *n* (%)	22 (81)
Diabetes mellitus, *n* (%)	17 (63)
Gastro-intestinal complaints, *n* (%)	15 (52)
Myopathy, *n* (%)	4 (15)
Muscular weakness, *n* (%)	4 (15)
Exercise intolerance, *n* (%)	14 (52)
Perceived fatigue, *n* (%)	10 (37)
Heteroplasmy levels blood, mean % (range)^b^	26 (8–62)
Heteroplasmy levels UEC, mean % (range)^c^	67 (30–93)
**NMDAS at screening, mean (range)**	23.1 (13–54)
**Diagnosis**	
CPEO	1 (3.7)
MELAS	1 (3.7)
MIDD	15 (55.6)
Mixed phenotype	10 (37.0)

Baseline characteristics of the 27 patients that entered the randomized controlled trial arm and received at least one dose of sonlicromanol (intent-to-treat population). For details of individual patients, see Supplementary Table 3. bid = twice daily; BMI = body mass index; CPEO = chronic progressive external ophthalmoplegia; MELAS = mitochondrial encephalopathy, lactic acidosis and stroke-like episodes; MIDD = maternally inherited diabetes mellitus and deafness; NMDAS = Newcastle Mitochondrial Disease Scale for Adults; RCT = randomized control trial; SD = standard deviation; UEC = urinary epithelial cells.

^a^Medical history data, i.e. medical conditions present prior to study medication initiation.

^b^ *n* = 22.

^c^ *n* = 21.

Patients who completed the RCT were eligible to enter the 52-week EXT and received sonlicromanol 100 mg bid. Re-evaluation against the in- and exclusion criteria for EXT acceptance was performed as a safety precaution because of the intrinsic progressive nature of m.3243A>G primary mitochondrial disease. Fifteen patients (62.5%; safety analysis set) agreed to participate in the EXT; these included 13 (86.7%) patients with MIDD, one (6.7%) with MELAS and one (6.7%) with MP. Twelve (80.0%) patients were included in the FAS (Fig. 1).

### Pharmacokinetics

#### Randomized controlled trial

The key pharmacokinetic parameters T_max_, C_max_ and AUC_last_ were measured at steady concentration and were consistent with the findings of previous studies, with either healthy volunteers or patients with the m.3243A>G mutation.^15,16^ A slightly more than proportional increase in the dose corrected C_max_ and exposure was seen; however, it was not significant (Supplementary Fig. 1A and B and Supplementary Table 4).

#### Open-label extension study

The compartmental models for sonlicromanol and KH183 derived from modelling the RCT pharmacokinetic values were applied to simulate the Week 52 exposure profile for the EXT (Supplementary Fig. 1C). Plasma concentrations were well predicted for predose and the 2- and 5-h post-dose samples and slightly overpredicted for the 30-min post-dose sample for sonlicromanol. For the metabolite (KH183), all the exposure measurements were well aligned with the model at all time points.

### Efficacy

#### Randomized controlled trial

**Figure 2:**
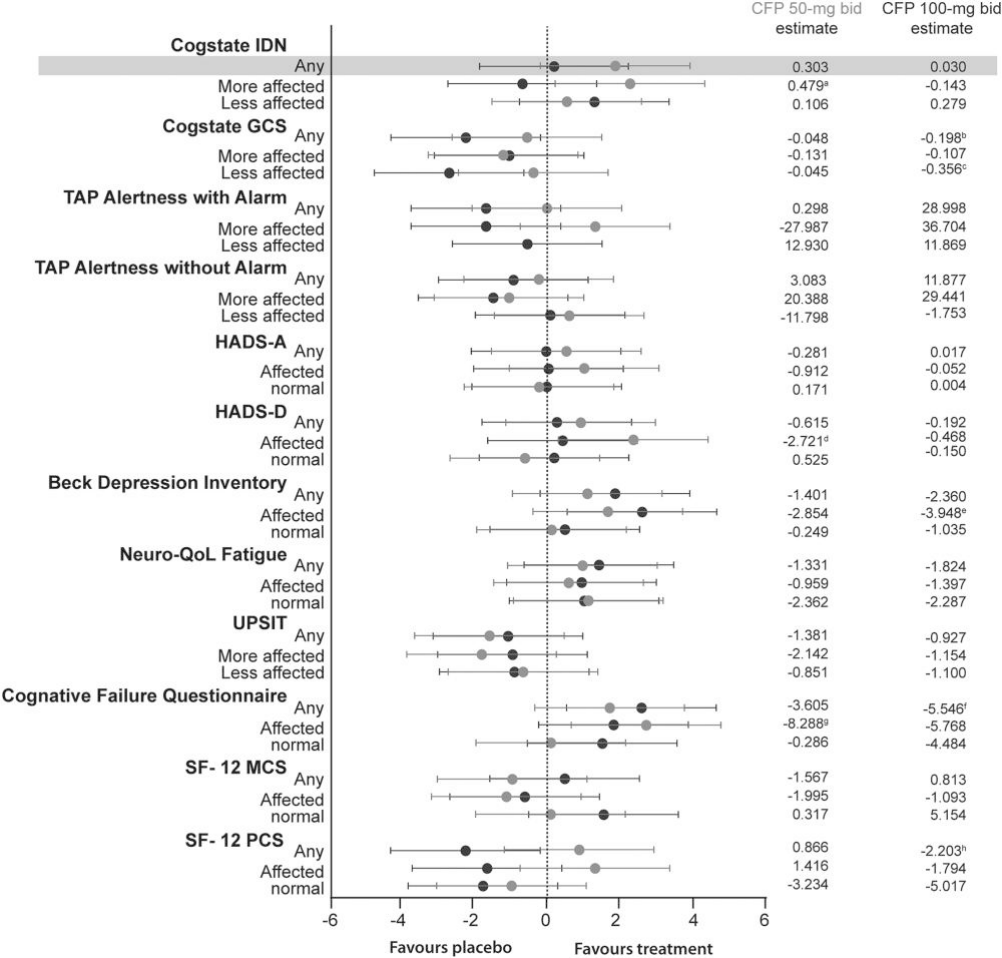
**Efficacy results of the randomized controlled trial**. *Left*: Forest plot of the treatment effects on all randomized controlled trial outcome measures; note that data from the headache and hearing assessments could not be presented in this manner because of data complexity. Treatment effect is the difference between the treatment means divided by the standard error of the difference. For all results, the treatment effect was adjusted so that in all cases a positive value represents an improvement of treatment versus placebo. Light grey indicates 50 mg twice daily (bid); dark grey, 100 mg bid. For the Cogstate visual identification (IDN) test, data from an extreme outlier were excluded. The Newcastle Mitochondrial Disease Adults Scale (NMDAS) score was not considered in this short-duration study. *Right*: Accompanying change from placebo (CFP) estimates. Significant *P*-values: ^a^0.034; ^b^0.035; ^c^0.012; ^d^0.026; ^e^0.014; ^f^0.015; ^g^0.011; ^h^0.034. Grey shading indicates the statistical analysis plan. GCS = Global Composite Score; HADS-A = Hospital Anxiety and Depression Scale-Anxiety; HADS-D = Hospital Anxiety and Depression Scale-Depression; MCS = Mental Component Score; PCS = Physical Component Score; TAP = Test of Attentional Performance; UPSIT = Pennsylvania Smell Identification Test.

Sonlicromanol did not produce a statistically significant improvement in attention performance in the primary model (Fig. 2, indicated with grey shading). When the model was adjusted for severity at period baseline per outcome measure, it showed a statistically significant response [0.479 (95% CI 0.040, 0.918); *P* = 0.034; 50 mg bid] compared with placebo in patients more severely affected in this outcome measure (Fig. 2).

To obtain exploratory data, all secondary outcome measures were analysed using this adjusted model without correction for multiple doses and outcome measures. This approach was used to find the best-responding outcome measures for an upcoming phase 3 trial.

All RCT outcome measures are described in Fig. 2 (forest plot of treatment effects and CFP estimates) and in more detail in Supplementary Table 5. Sonlicromanol treatment showed improvement in the HADS-D scores in the more affected at baseline patients [50 mg bid: −2.7209 (95% CI −5.081, −0.361); *P* = 0.026]. The HADS-A scores did not show similar effects in either dose group, although similar numbers of patients were affected in each of the HADS subscales (anxiety and depression) (Supplementary Table 2). Consistent with the HADS-D scores, the BDI scores also showed significant improvement, with estimates of up to −2.9 points (95% CI −6.3989, 0.690; *P* = 0.110) in the 50 mg bid and −3.9 points (95% CI −7.038, −0.859; *P* = 0.014) in the 100 mg bid-treated patients more affected in this outcome measure (approximately 50% of patients were affected at predose) (Supplementary Table 2).

A positive effect on cognition levels was observed in CFQ scores [100 mg bid estimate −5.55 (95% CI −9.893, −1.199); *P* = 0.015]. A stronger effect on CFQ scores was observed in the patients more affected at baseline for this outcome measure [50 mg bid: −8.29 (95% CI −14.531, −2.046); *P* = 0.011; 100 mg bid: −5.58 (95% CI −12.209, 0.673); *P* = 0.077]. The Cogstate Global Composite Score, a combination of several of the subdomains, showed an effect of the placebo only in patients less affected in this outcome measure that could not be explained. Other outcome measures did not show changes with *P* < 0.05 in the RCT study, as shown in Fig. 2 and Supplementary Table 5.

#### Open-label extension study

In the EXT, CFB data were available for 11 patients (Table 2). Patients treated for 52 weeks with 100 mg bid sonlicromanol did not improve their attention performance (Cogstate IDN).

**Table 2:** Observed treatment effects (open-label extension study)

PD values					CFB 52 weeks					
					Paired *t*-test				Responder analysis	
Outcome measure	Subscore	*n*	Affected	Mean (SD)	*n*	Mean (SD)	*P*-value	95% CI	total responders	Responding > MCID
Cogstate	IDN	12	8	−1.048 (1.018)	11	0.277 (0.555)	0.129	−0.0963 to 0.6496	7	3
BDI		12	7	10.8 (7.6)	11	−4.5 (6.8)	0.056	−9.1 to 0.1	10	9
CFQ		12	3	31.9 (13.2)	11	−4.6 (7.4)	0.065	−9.6 to 0.3	7	4
NQF		12	7	52.5 (5.4)	11	−6.3 (5.5)	**0.004**	−10.00 to −2.58	9	5
TAP	Without alarm	12	–	312.3 (55.0)	11	−34.2 (31.3)	**0.005**	−55.2 to −13.1	9	–
TAP	With alarm	12	–	322.6 (69.7)	11	−56.6 (59.5)	**0.010**	−96.6 to −16.7	10	–
SF-12	PCS	12	11	37.9 (9.0)	11	7.7 (5.4)	**0.0008**	4.1 to 11.4	10	8
MPQ		12	12	10.5 (5.8)	10	−5.7 (5.6)	**0.011**	5.5 to 32.3	10	4
Rand SF36	Pain	12	10	55.6 (20.2)	11	18.9 (20.0)	**0.011**	5.5 to 32.3	8	8
NMDAS		12	12	21.4 (8)	11	−1.5 (3.1)	0.130	−3.6 to 0.5	6	6^a^
EQ-5D	VAS	12	12	61.7 (15.6)	10	16.2 (18.4)	**0.021**	3 to 29.4	7	7
EQ-5D	Index value	12	–	0.793 (0.1262)	11	0.074 (0.085)	**0.017**	0.016 to 0.131	10	–
Five Times SST		11	11	13.75 (3.02)	11	−1.21 (2.13)	0.088	−2.64 to 0.22	7	3
mini-BESTest		10	6	0.778 (0.1820)	9	0.087 (0.051)	**0.0009**	1.3 to 3.5	8	3

Of the indicated outcome measures (or subscores) the values before entering the open-label extension study (EXT) are indicated [predose (PD; baseline); *n* (number of affected patients); and mean and standard deviation]. After 52 weeks of treatment, the change from baseline (CFB) was analysed in a paired *t*-test wherein *n* (number of paired data-points), mean CFB, SD and *P*-values (in bold when *P* < 0.05) were calculated. No correction for the multitude of outcome measures was done for any of the analyses. In the responder analysis the number of patients responding in the direction of improvement was scored, as well as the number of patients with a CFB > MCID (minimal clinically important difference). For additional data see Supplementary Table 6. BDI = Beck Depression Inventory; CFQ = Cognitive Failure Questionnaire; CI = confidence interval; EQ-5D = EuroQol 5-Dimensions; IDN = visual identification task; mini-BESTest = mini-Balance Evaluation Systems test; MPQ = McGill Pain Questionnaire; NQF = Neuro-Quality of Life Short Form-Fatigue scale; NMDAS = Newcastle Mitochondrial Disease Adults Scale; PCS = Physical Component Score; SF-12 = 12-item short form survey; SF-36 = 36-item short form survey; SST = Sit-to-Stand Test; TAP = Test of Attentional Performance; VAS = Visual Analog Scale.

^a^Any improvement in NMDAS was regarded as clinically meaningful.

**Figure 3:**
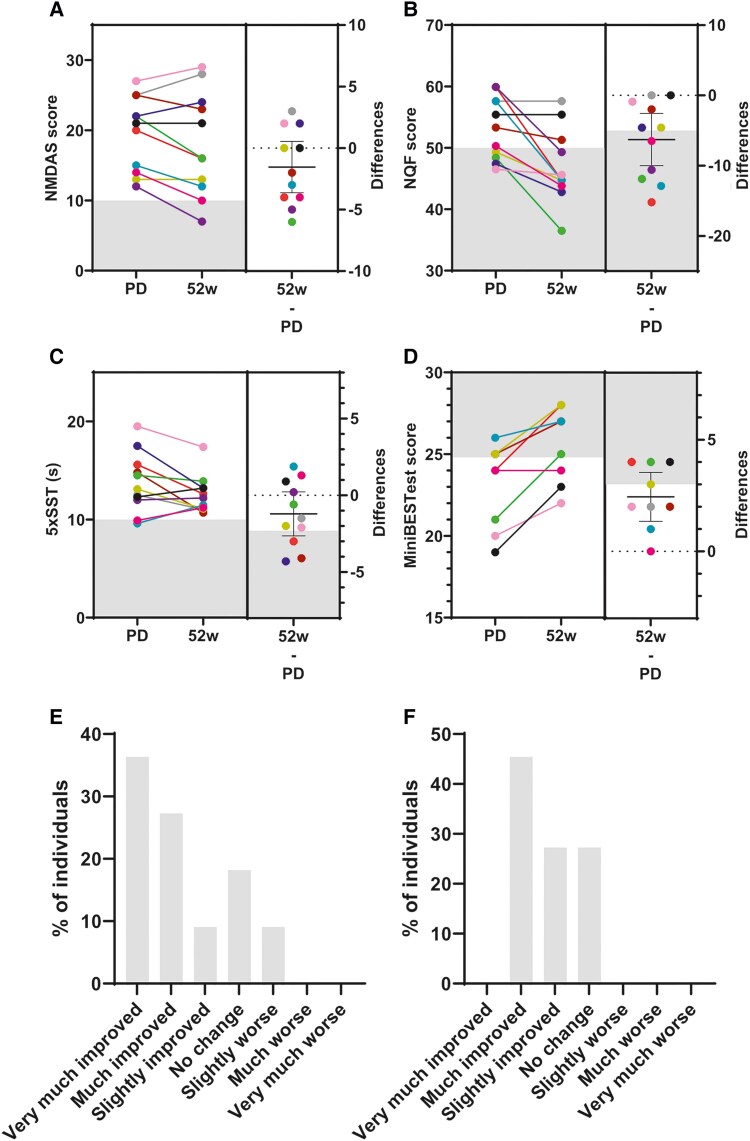
**Health improvements following 52 weeks of sonlicromanol treatment**. (**A**–**D**) *Left*: Individual scores on the indicated measures during the 52 weeks of sonlicromanol treatment. The grey-shaded area depicts the normal ranges. *Right*: The grey-shaded area shows the threshold greater than the minimal clinically important difference (MCID). The dots represent the change from baseline (CFB) for each patient. The dotted line represents no change. (**A**) Newcastle Mitochondrial Disease Scale for Adults (NMDAS); (**B**) Neuro-Quality of Life Short Form-Fatigue (NQF); (**C**) Five Times Sit-to-Stand Test (5×SST); (**D**) mini-Balance Evaluation Systems test (mini-BESTest); (**E**) Patient-reported Global Impression of Change (PGIC); (**F**) Clinician-scored Global Impression of Change (CGIC). PD = predose.

Changes observed in both the BDI and CFQ in the RCT were confirmed in the EXT. Longer-term treatment showed an average improvement in the BDI of −4.5 points (95% CI −7.0, 1.0; *P* = 0.0563). Ten of the eleven patients showed an improvement in the BDI (7/12 patients were affected at predose). Nine patients had improvements more than the MCID. The mean CFB for the CFQ was −4.6 points (95% CI −9.0, 1.0; *P* = 0.0650); only three patients were affected at predose. The long-term treatment effect of sonlicromanol was more obvious for several outcome measures. In the RCT, the NQF did not show a significant improvement over the placebo. However, in the EXT, the CFB of −6.29 points (95% CI −11.90, −0.90; *P* = 0.0036) was much greater than the placebo effect observed in the RCT (−0.42 points), and treatment effects were observed throughout the course of the EXT (Fig. 3B).

Relevant improvements were also observed in TAP without alarm [CFB −34.2 (95% CI −64.0, −4.0); *P* = 0.0047] and with alarm [CFB −56.6 (95% CI −107.0, −5.0); *P* = 0.0102] in the EXT; these were not significant in the earlier RCT study.

The PCS part of the SF-12 self-reported outcome of everyday life showed improvement in the EXT [CFB 7.7 (95% CI 2.9, 9.5); *P* = 0.0008]. Eight patients had improvements in PCS greater than the MCID of five points. However, the mental component score (MCS) did not show any changes. The repeated measures ANCOVA (Supplementary Table 6) showed similar results for these outcome measures, indicating that these improvements were observed throughout the different time points.

Several other outcome measures also showed an improvement in the EXT. Patients experienced decreased pain as evidenced by reduced MPQ scores [−5.7 points (95% CI −7.0, −2.0); *P* = 0.0105]; four patients showed improvements greater than the MCID. Consistent with this, the RAND SF-36 health survey for pain also demonstrated improvements [18.9 (95% CI 0.0, 32.0); *P* = 0.0105], with eight patients showing changes greater than the MCID. Other domains of the SF-36 also demonstrated improvements in the EXT (Supplementary Table 6). Improvements were also seen in the NMDAS scores (Fig. 3A), with 11 patients showing an average decrease of 1.5 points (95% CI −4.0, 2.0; *P* = 0.1304). General disease improvement was also observed in CGIC and PGIC scale scores (Fig. 3E and F). Most patients responded well on both the EQ-5D-5L VAS scale [*n* = 7, all greater than MCID; CFB: 16.2 (95% CI 0.0, 30); *P* = 0.0213] and the Index value [*n* = 10; CFB: 0.0737 (95% CI 0.0390, 0.0930); *P* = 0.0173].

The performance-based outcome measures evaluated at 52 weeks showed positive signals in the EXT study. The Five Times Sit-to-Stand Test showed an average decrease of 1.21 s (95% CI −3.00, 0.90; *P* = 0.088), with improvements seen in 7 of 10 patients who were in the affected range (Fig. 3C). The MCID used for the Five Times Sit-to-Stand Test is the most conservative MCID known in the literature (−2.3 s), yet three patients showed improvement larger than the MCID.^36^ The dynamic balance of the patients, assessed using the mini-BESTest, also improved [2.4 (95% CI 1.4, 1.3), *P* = 0.0009; see also Fig. 3D]. The handgrip strength of the non-dominant hand showed an increase in muscle strength; four patients showed improvement exceeding the MCID (5 kg). No improvements were observed in the sense of smell and hearing assessed using the UPSIT and PTA scales, respectively.

### Dose selection

**Figure 4:**
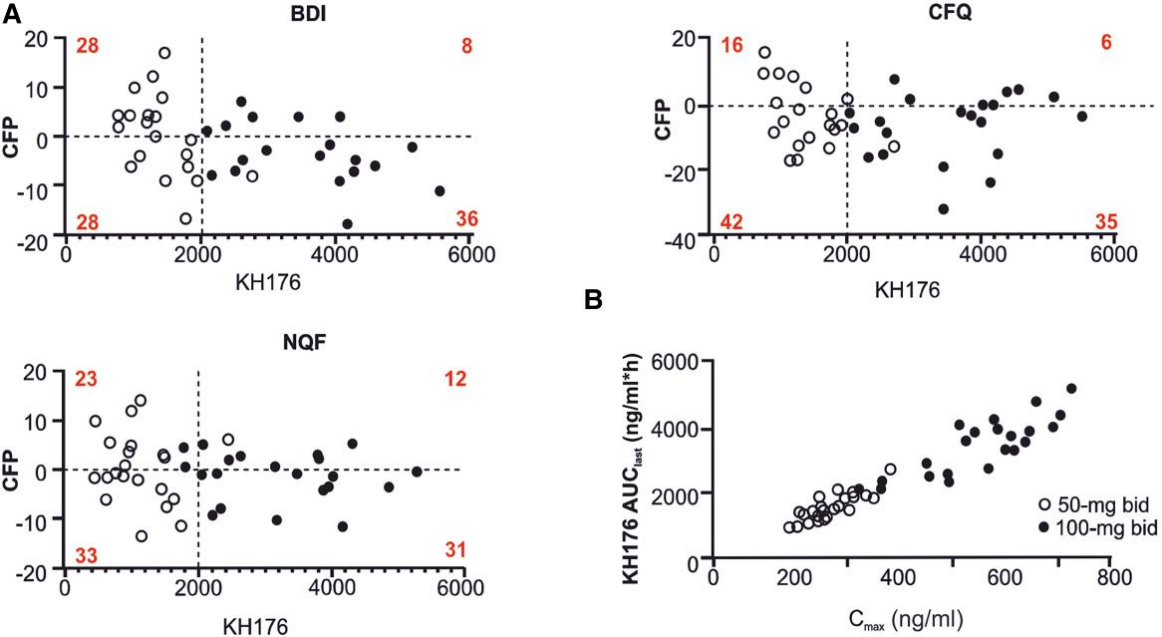
**Dose selection**. (**A**) Individual Beck Depression Inventory (BDI), change from placebo (CFP) and Neuro-Quality of Life Short Form-Fatigue (NQF) values were obtained from outcome measures of the randomized controlled study and showed positive trends toward improvement. They were plotted against the plasma concentration of KH176 (parent compound) for both the 50-mg twice daily (bid) and 100-mg bid doses. The four quadrants of each graph represent positive (*top*)/negative (*bottom*) responses and more (*right*)/less (*left*) than 2000 ng/ml/h. Numbers denoted in red refer to the percentage of points scored. (**B**) A correlation between the individual maximum plasma concentration (C_max_) and the exposure data for the 50-mg bid and 100-mg bid doses. CFQ = Cognitive Failure Questionnaire.

Optimal dose selection was determined by a comparative dose-exposure-response analysis (Fig. 4A). The individual CFP values of outcome measures that showed the most positive trends in the RCT were plotted against the plasma concentration of KH176 for both the 50-mg bid and 100-mg bid doses. This plot was then divided into four quadrants of positive/negative responses and more/less than 2000 ng/ml/h (lowest exposure observed for the 100 mg bid). For each quadrant, the percentage of points scored was calculated. Using this analysis for the NQF, BDI, CFQ and SF-12 PCS, an exposure >2000 ng/ml resulted in a better ratio between responding and non-responding scores. To illustrate, for BDI, an exposure <2000 ng/ml/h showed responding scores (28%) comparable to non-responding scores (28%), whereas an exposure >2000 ng/ml/h resulted in higher responding scores (36%) than non-responding scores (8%). The 100-mg bid dose did not result in C_max_ concentrations higher than 1000 ng/ml (Fig. 4B), a threshold potentially causing safety concerns.^16^

### Safety

#### Randomized controlled trial

Most adverse events were classified as mild or moderate, with no records of any severe treatment-emergent adverse events (TEAEs) (Table 3 and Supplementary Table 7) or suspected unexpected serious adverse reactions (SUSARs). No dose-dependent increases in Fridericia-corrected QT interval (QTcF) and QTc increases >60 ms were observed. One patient receiving placebo and one patient treated with sonlicromanol 100 mg bid showed one recorded increase of QTc by 32 ms and 36 ms, respectively (QTc = 431 ms). In both cases, the QTc remained within normal limits (defined as ≤450 ms for males and ≤470 ms for females). Three patients experienced adverse events resulting in study discontinuation. These included one patient being withdrawn due to an allergic skin reaction to the medical device (Holter pads) after taking the first dose of sonlicromanol 50 mg during treatment period 1 (TP1) of the RCT. This patient had mild signs of Holter pad (Medi-Derma S and Skintact) skin reaction during the screening assessments, and the reaction worsened at baseline. The second patient was discontinued from treatment at the end of treatment period 2 (TP 2) and did not receive placebo due to a T-wave depression (Days 28–43) after a period of drug interruption due to palpitations, chest pain, disturbance in attention, altered state of consciousness, memory impairment (all symptoms for Days 19–52) and decreased exercise tolerance (Days 19–55). Further cardiological assessments, including cardiac screening blood tests and coronary angiography revealed normal results. The third patient was discontinued from treatment due to abnormal T-wave on ECG following the first dose of sonlicromanol 100 mg bid in treatment period 3 (TP3). This event was considered unrelated to treatment, as it was the first dose after 22 days of a drug-free period and 3 days post coronavirus disease 2019 (COVID-19) vaccination. No other cardiological adverse events were detected.

**Table 3:** Sonlicromanol safety profile

Patients with TEAE, *n* (%)	RCT			EXT
	Placebo (*n* = 25)	50 mg bid (*n* = 27)	100 mg bid (*n* = 26)	100 mg bid (*n* = 15)
Patients with at least one TEAE	15 (60)	14 (52)	12 (46)	13 (87)
Drug-related TEAE	5 (20)	7 (26)	7 (27)	8 (53)
Serious TEAE	0 (0)	0 (0)	0 (0)	2 (13)
Drug-related serious TEAE	0 (0)	0 (0)	0 (0)	1 (7)^a^
Permanent discontinuation of study drug due to TEAE	0 (0)	1 (4)^b^	1 (4)^c^	0 (0)
Drug-related TEAE leading to permanent discontinuation of study drug	0 (0)	0 (0)	0 (0)	0 (0)
Patients with at least one severe TEAE	0 (0)	0 (0)	0 (0)	2 (13)^d^

Undesirable events not present prior to medical treatment, or an already present event that worsens either in intensity or frequency following the treatment. bid = twice daily; EXT = open-label extension study; RCT = randomized controlled trial; TEAE = treatment-emergent adverse event; TP1D1 = treatment period 1 Day 1; TP3DI = treatment period 3 Day 1.

^a^Addison crises triggered due to infection.

^b^TP1D1 allergy to Holter pads.

^c^TP3D1 22 days after last SM; patient received COVID-19 Vaxzevria vaccine.

^d^Two patients reported severe TEAEs following sonlicromanol, including acute adrenocortical insufficiency, and in one patient retinal detachment, foot fracture, internal limiting membrane peeling, medical procedure and deep vein thrombosis. All severe TEAEs were considered unlikely to be related to the study medication.

#### Open-label extension study

Most adverse events in the EXT were mild to moderate. Two patients had severe TEAEs (Table 3). One patient with polyglandular autoimmune syndrome type 2 reported two serious TEAEs: two episodes of Addison's crisis, triggered due to infection. However, there were no discontinuations from the study in the EXT.

## Discussion

The results of a phase 2b clinical program in m.3243A>G primary mitochondrial disease patients are reported. The program consisted of a three-way, cross-over RCT and a 1-year EXT study evaluating the pharmacokinetics, safety and efficacy of sonlicromanol. A broad selection of outcome measures was evaluated to make an educated selection for the outcome measures to be included in the phase 3 trial.

The pharmacokinetic results obtained in this study (C_max_ of 500 ng/ml and T_max_ between 1 and 2 h) are consistent with the earlier pharmacokinetic findings in both healthy volunteers and patients with mitochondrial DNA tRNA^Leu[UUR]^ m.3243A>G mutation.^15,16^ Since hERG (human ether-a-go-go-related gene) channel inhibition occurred in the earlier phase 1 study at high drug exposures only,^16^ a cautious approach was adopted in the earlier phase 2a study as well as this phase 2b program, wherein QTc prolongation was repeatedly monitored in all the patients (>1000 ECGs and >700 days of Holter evaluations). At the dosing applied in the current program, there was no QTc prolongation observed, thereby confirming the long-term safety of sonlicromanol. An ongoing study, wherein a subset of these patients who were followed up to 78 weeks and subsequently entered the named patient program of 6 months, further confirmed the long-term tolerability and safety of sonlicromanol (data on file). Comparative dose-exposure-response analysis indicated that 100 mg bid was the optimal sonlicromanol dose in adult patients (>18 years old).

The cognitive domain of attention (primary end point RCT) using the Cogstate IDN test was chosen to obtain more quantifiable results than the cognitive domain of the TAP that had shown effectiveness in the phase 2a study.^15^ However, in this phase 2b RCT, the Cogstate IDN test did not achieve statistical significance in either treatment group (50 and 100 mg bid) in the unadjusted model (mixed-model analysis). This may be attributed to large variability in the scores present at baseline and the inherent variable multi-systemic nature of the population with the m.3243A>G mutation. However, the primary end point showed a positive trend towards improvement after adjusting for the baseline, especially in more severely affected patients. While it was difficult to obtain any significant improvement in the cognitive domain of attention in the patients in the EXT due to the small sample size, most patients showed a positive CFB.

The modified mixed-model analysis of the BDI showed significant improvements in patients who were more severely affected, following 100 mg bid treatment with sonlicromanol. In the EXT, most patients showed an improvement greater than the MCID threshold for the BDI score. These results are consistent with the findings of the earlier phase 2a study, demonstrating significantly improved BDI scores in patients treated with sonlicromanol.^15^ Thus, the BDI can be considered a useful outcome measure for determining mood changes in patients with m.3243A>G mutation.

The CFQ score, which assessed the effect of sonlicromanol on cognitive failure, indicated a large improvement, especially in patients treated with 100 mg bid compared to placebo in the unadjusted model. In the baseline-adjusted model, affected patients treated with 50 mg bid improved significantly. This dose-variability effect may be attributed to the low number of patients with CFQ scores in the affected range at predose (Supplementary Table 2).

A similar result was shown in the EXT, where only two of six patients were affected on the CFQ scale at predose, resulting in an average decrease of 4.6 points. This finding was not statistically significant due to the large range of responses. The TAP tests showed significant improvement in the EXT compared with baseline; however, the lack of a placebo group makes further interpretation of the results difficult. Moreover, there was a high degree of variability in the TAP test results in the RCT; thus, positive results may be overestimated.

Apart from clinical heterogeneity and the low number of patients encountered with mitochondrial diseases, one of the major challenges in treatment development for mitochondrial diseases is the lack of validated outcome measures and their use in natural history studies and intervention studies. Certain measures have been applied in intervention studies, but very few have been validated in patients with mitochondrial disease.^37-40^ The NMDAS score is one such validated clinical outcome measure and a widely applied test that assesses disease severity in this patient population used in natural history studies.^41^ The NMDAS score improved in 6 of 11 patients by up to 6 points (Fig. 3A), and this improvement was seen to increase slowly over time. All these patients received sonlicromanol 100-mg bid treatment for 52 weeks. The NMDAS score remained stable in one patient. Relative to the expected annual increase in NMDAS score (deterioration) of 1.31 points for MELAS and 0.64 points for MIDD per year, as found in the previously conducted 6-year prospective natural history study in patients with m.3243A>G mutation, this decline in the NMDAS score implies a substantial decrease in the severity of the disease.^41^ In primary mitochondrial myopathies, the expected annual NMDAS increase was found to be 0.9 points per year.^42^

Other outcome measures (SF-12 PCS, TAP tests and NQF) also showed much improved results in the EXT versus the RCT. This indicated that the shorter study duration of 28 days may be important for assessing the initial tolerability, safety, pharmacokinetics and signal seeking but is most likely not indicative of the expected long-term effects. There was no treatment effect observed for the PTA hearing test and the UPSIT smell test; this may be attributed to the death of sensory cells.

The NQF CFB in the EXT study (LS mean = −6.3) was much greater than the placebo effect measured in a recent 24-week study in patients with primary mitochondrial myopathies (LS mean = −2.62).^38^ A strong correlation was observed between different outcome measures of similar domains like pain severity (RAND SF-36 and MPQ) and fatigue (NQF and energy/fatigue domain RAND SF-36) and an improvement in disease severity (EQ-5D-5L scales, several RAND SF-36 domains, PGIC and NMDAS). These observations add additional value to the efficacy of the observed data, but it is challenging to ascertain the treatment effects of sonlicromanol without a placebo group attribution of the results.

Among the 24 eligible patients in the RCT, most chose to be enrolled in the EXT (*n* = 18; 15 dosed plus three screen failures). These 15 patients had variable RCT treatment responses. The effects of sonlicromanol treatment are considered systemic, since improvements were found in the domains frequently affected by primary mitochondrial disease such as the brain (NQF, CFQ, RAND SF-36 domains) and muscles (mini-BESTest, Five Times Sit-to-Stand Test).

In conclusion, the selected dose of 100 mg bid sonlicromanol was found to be safe and well tolerated for up to 52 weeks, without showing clinically relevant QTc prolongation. Furthermore, the results of the phase 2b program provide insights for the selection of end points in a larger 52-week phase 3 RCT study in more homogeneous, more severely affected m.3243A>G patients.

## Supplementary Material

awae277_Supplementary_Data

## Data Availability

The authors confirm that the data supporting the findings of this study are available within the article and its supplementary material. Upon reasonable request, raw data that support the findings of this study can be made available by the corresponding author.
