# Supplementary material for: Phase 2b program with sonlicromanol in patients with mitochondrial disease due to m.3243A>G mutation
Source: Brain. 2024 Nov 6;148(3):896–907. doi: 10.1093/brain/awae277 (PMC11884763; doi:10.1093/brain/awae277)
Supplement: awae277_Supplementary_Data [file awae277_supplementary_data.pdf]

Supplementary Appendix

Inclusion And Exclusion Criteria

Inclusion Criteria

1. Males and females aged 18 years or older at screening.
2. Ability and willingness to provide written Informed Consent prior to screening evaluations.
3. Confirmed mitochondrial DNA $tRNA^{Leu(UUR)}$ m.3243A>G mutation.
4. Positive NMDAS score >10 at Screening.
5. Three or more clinical features, with no other causative unifying diagnosis, found to commonly occur in subjects with an m.3243A>G mutation:
   - Deafness
   - Developmental delay
   - Diabetes Mellitus
   - Epilepsy
   - Gastrointestinal complaints
   - Progressive External Ophthalmoplegia (PEO) and retinopathy
   - Ataxia
   - Exercise intolerance
   - Fatigue
   - Migraine (with or without aura), specified by at least five attacks fulfilling diagnostic criteria B-D:

     B. Headache attacks lasting 4-72 hours (untreated or unsuccessfully treated)

     C. Headache has at least two of the following four characteristics:

     1. unilateral location
     2. pulsating quality
     3. moderate or severe pain intensity
     4. aggravation or causing avoidance of routine physical activity (e.g. walking or

climbing stairs)

D. During headache at least one of the following:

1. nausea and/or vomiting
2. photophobia and phonophobia

6. Attentional dysfunction score (Cogstate Identification test) ≥ 0.5 standard deviations poorer than healthy controls at Screening.
7. Disease appropriate physical and mental health as established at Screening by medical history, physical examination, ECG, and vital signs recording, and results of clinical chemistry and haematology testing as judged by the investigator.
8. Objectified Left Ventricular Ejection Fraction (LVEF) ≥45% (echocardiography, or otherwise).
9. Left Ventricular (LV) wall thickness ≤15 mm.
10. Left atrium dilatation ≤ 40 mL/m^2.

    Note: No need to test LV parameters (criteria #8, #9, #10) if favourable echocardiography (or otherwise) results dated less than 6 months prior to Screening are available.

11. Women of childbearing potential must be willing to use highly effective contraceptive methods during the entire study, i.e., combined (estrogen and progestogen containing) oral, intravaginal or transdermal hormonal contraception associated with inhibition of ovulation;, oral, injectable or implantable progestogen-only hormonal contraception associated with inhibition of ovulation; use of an intrauterine device; an intrauterine hormone releasing system, bilateral tubal occlusion and vasectomy of the partner.

    Any hormonal contraception method must be supplemented with a barrier method (preferably male condom).

    Vasectomised partner is considered a highly effective birth control method if partner is the sole sexual partner of the subject and the vasectomised partner has received a medical assessment of the surgical success. Sexual abstinence is considered a highly effective method only if defined as refraining from heterosexual intercourse during the entire period of risk associated with the study treatments. The reliability of sexual abstinence needs to be evaluated in relation to the duration of the clinical trial and the preferred and usual lifestyle of the subject. Periodic abstinence (e.g.,

calendar, ovulation, symptothermal, post-ovulation methods) and withdrawal are not acceptable methods of contraception.

Note 1: Natural family planning methods, female condoms, cervical cap, or diaphragm are not considered adequate contraceptive methods in the context of this study.

Note 2: To be considered not of childbearing potential, potential female subjects must be post-menopausal for at least two years, or have been surgically sterilised (bilateral tubal ligation, hysterectomy or bilateral oophorectomy) for at least 6 months prior to screening.

Note 3: KH176 has been shown non-genotoxic judged from the Ames test, Chromosomal Aberration test, and *in vivo* Micronucleus test. Moreover, appreciable systemic exposure from the exposure to (~2.5 mL) semen is extremely unlikely. However, until reproductive toxicology studies have confirmed that KH176 does not adversely affect normal reproduction in adult males and females, as well as causing developmental toxicity in the offspring, the following contraceptive precautions must be adhered to:

- male subjects with female partners of childbearing potential must be willing to use condoms during the entire study.
- female partners of childbearing potential of male subjects must be willing to use adequate contraceptive methods during the entire study, i.e., a hormonal contraceptive method (pill, vaginal ring, patch, implant, injectable, hormone-medicated intrauterine device) or an intrauterine device.

12. Able to comply with the study requirements, including swallowing study medication.

Exclusion Criteria

1. Surgery of gastrointestinal tract that might interfere with absorption.
2. Treatment with an investigational product within 3 months or 5 times the half-life of the investigational product (whichever is longer) prior to the first dose of the study medication.
3. Documented history of ventricular tachycardia (HR>110 beats/min).

4. History of acute heart failure, (family) history of unexplained syncope or congenital long and short QT syndrome or sudden death.
5. Clinically relevant abnormal laboratory, vital signs or physical or mental health.
   a. Aspartate aminotransferase (ASAT) or alanine aminotransferase (ALAT) > 3 x upper limit of normal (ULN), or bilirubin > 3 x ULN at screening. If a patient has ASAT or ALAT > 3 x ULN but < 3.5 x ULN, re-assessment is allowed at the investigator's discretion.
   b. Estimated glomerular filtration rate ≤ 60 mL/min according to the CKD-EPI formula at screening.
   c. Systolic Blood pressure > 150 mmHg at screening or baseline.
   d. All other clinically relevant parameters at screening or baseline as judged by the Investigator.
6. Clinically relevant abnormal ECG or cardiac functioning, defined as ST-segment elevation > 1 mm in I, II, III, aVL, aVF ,V3 ,V4 ,V5 ,V6; > 2 mm in V1, V2; QTc > 450 ms for male subjects; QTc: > 470ms for female subjects (local, machine read), T-top inversion in >1 consecutive lead.
7. Serum Hyper-potassium (> 5.0 mEq/L).
8. Serum Hypo-potassium (< 3.5 mEq/L).
9. History of ischemic heart disease.
10. Symptomatic heart failure.
11. Clinically relevant aorta and/or mitralis valvular defect as judged by the investigator.
12. Pregnancy or breast feeding (females).
13. Poor nutritional state as judged by the investigator.
14. History of hypersensitivity or idiosyncrasy to any of the components of the investigational drug.
15. Medical history of drug abuse (illegal drugs such as cannabinoids, amphetamines, cocaine, opiates, or problematic use of prescription drugs such as benzodiazepines, opiates).
16. The use of any of the following medication and/or supplements within 4 weeks or 5 times the half-life (whichever is longer) prior to the first dosing of the study medication:

a. (multi)vitamins, co-enzyme Q10, Vitamin E, riboflavin, and anti-oxidant supplements (including, but not limited to idebenone/EPI-743, mitoQ); unless stable for at least one month before first dosing and remaining stable throughout the study.

b. any medication negatively influencing mitochondrial functioning (including but not limited to valproic acid, glitazones, statins, anti-virals, amiodarone, and non-steroidal anti-inflammatory drugs (NSAIDs)), *unless* stable for at least one month before first dosing and remaining stable throughout the study.

   Note: thus, mitoQ and any medication negatively influencing mitochondrial functioning are allowed as long as the dose has been stable for at least one month prior to first dosing and remains stable throughout the study.

c. any strong Cytochrome P450 (CYP)3A4 inhibitors (all 'conazoles-anti-fungals', HIV antivirals, grapefruit).

d. strong CYP3A4 inducers (including HIV antivirals, carbamazepine, phenobarbital, phenytoin, rifampicin, St. John's wort, pioglitazone, troglitazone).

e. any medication known to affect cardiac repolarisation, *unless* the QTc interval at screening is normal during stable treatment (all anti-psychotics, several anti-depressants, e.g. nor/amitriptyline, fluoxetine, anti-emetics: domperidone (motilium®) granisetron, ondansetron). For a complete list see https://crediblemeds.org.

f. any medication metabolised by CYP with a narrow therapeutical width. For reference (Germany and United Kingdom): drug interaction table of Indiana University (http://medicine.iupui.edu/clinpharm/ddis/clinical-table/). For reference (The Netherlands): KNMP Kennisbank (https://www.knmp.nl/producten/knmp-kennisbank/inloggen-knmp-kennisbank. For reference (all other countries): drug interaction table of Indiana University (http://medicine.iupui.edu/clinpharm/ddis/clinical-table/).

Non-compartmental analysis of pharmacokinetics (NCA)

$AUC_{0\text{-}tau}$ was calculated using the linear-log trapezoidal method (linear up, log down). $AUC_{0\text{-}tau}$ was calculated from the dosing time until the end of the nominal dosing interval (12 hours). If the latter time point was not available, it was imputed via logarithmic extrapolation of the regression slope λ_z. The terminal slope was estimated by linear regression of the logarithmically transformed concentration versus time data. The terminal elimination rate constant was not determined if only two or fewer data points were available. In case λ_z was unavailable, no interpolation could be done for $AUC_{0\text{-}tau}$, and $AUC_{0\text{-}tau}$ was not calculated if interpolation was needed. $AUC_{0\text{-}tau}$ values for which the following criteria were not met were considered with caution:

- the percentage of extrapolated AUC exceeded 20%,
- fewer than three different time points were used to calculate the terminal phase, and
- the coefficient of determination adjusted R^2 was smaller than 0.85.

In these cases, the $AUC_{0\text{-}tau}$ values were excluded from descriptive statistics, unless they were deemed reliable by the pharmacokinetics that performed the analysis.

PK data was collected for 26 patients as one subject dropped out from the study very early. The KH176 and KH183 PK profiles were not measured for three patients in the 100-mg bid treatment and two patients in the 50 mg bid treatment. One patient exhibited an implausible PK profile for the 50-mg bid treatment and was therefore not included in the NCA.

In total, the evaluable PK analysis population comprised 23 patients for the 50-mg bid treatment and 23 patients for the 100-mg bid treatment.

References Supplemental Material:

Angst F, Aeschlimann A, Stucki G (2001). Smallest detectable and minimal clinically important differences of rehabilitation intervention with their implications for required sample sizes using WOMAC and SF-36 quality of life measurement instruments in patients with osteoarthritis of the lower extremities. *Arthritis & Rheumatism*, *45*(4), 384–391.

Broadbent DE, Cooper PF, FitzGerald P, Parkes KR. (1982). The cognitive failures questionnaire (CFQ) and its correlates. *British Journal of Clinical Psychology*, *21*(1), 1–16.

Button KS., Kounali D, Thomas L, Wiles NJ, Peters TJ, Welton NJ, Ades AE, Lewis G. (2015). Minimal clinically important difference on the Beck Depression Inventory-II according to the patient's perspective. *Psychological Medicine*, *45*(15), 3269–3279.

Dahl HHM, Thorburn DR. (2001). Clinical spectrum and diagnosis of mitochondrial disorders. *American Journal of Medical Genetics - Seminars in Medical Genetics*, *106*(1), 4–17.

De Laat P, Rodenburg RR, Roeleveld N, Koene S, Smeitink JA., Janssen MCH. (2021). Six-year prospective follow-up study in 151 carriers of the mitochondrial DNA 3243 A>G variant. *Journal of Medical Genetics*, *58*(1), 48–55.

Hawker GA, Mian S, Kendzerska T, French M. (2011). Measures of adult pain - Visual Analog Scale for Pain (VAS Pain), Numeric Rating Scale for Pain (NRS Pain), McGill Pain Questionnaire (MPQ), Short-Form McGill Pain Questionnaire (SF-MPQ), Chronic Pain Grade Scale (CPGS), Short Form-36 Bodily Pain Scale (SF-36 BPS), and Measure of Intermittent and Constant Osteoarthritis Pain (ICOAP). *Arthritis Care and Research*, *63*(SUPPL. 11).

Herrmann, C. (1997). International experiences with the Hospital Anxiety and Depression scale - a review of validation data and clinical results. *Journal of Psychosomatic Research*, *42*, 1741.

Huber M, Reitmeir P, Vogelmann,M, Leidl R. (2016). EQ-5D-5L in the General German Population: Comparison and Evaluation of Three Yearly Cross-Section Surveys. *International Journal of Environmental Research and Public Health*, *13*(3), 343.

Janssen MF, Szende A, Cabases J, Ramos-Goñi JM, Vilagut G, König HH. (2019). Population norms for the EQ-5D-3L: a cross-country analysis of population surveys for 20 countries. *The European Journal of Health Economics*, *20*(2), 205–216.

Jensen MB., Jensen CE, Gudex C, Pedersen KM, Sørensen SS, Ehlers LH. (2023). Danish population health measured by the EQ-5D-5L. *Scandinavian Journal of Public Health*, *51*(2), 241–249.

Klukowksa AM, Staartjes VE, Vandertop WP, Schröder ML. (2021). Five-repetition sit-to-stand test performance in healthy individuals: reference values and predictors from 2 prospective cohorts. *Neurospine, 18(4),*760-769.

Meretta BM, Whitney SL, Marchetti GF, Sparto PJ, Muirhead RJ. (2007). The five times sit to stand test: Responsiveness to change and concurrent validity in adults undergoing vestibular rehabilitation. *J Vestibular Res*, *16*(4–5), 233–243.

Nowinski CJ, Siderowf A, Simuni T, Wortman C, Moy C, Cella D. (2016). Neuro-QoL health-related quality of life measurement system: Validation in Parkinson's disease. *Movement Disorders*, *31*(5), 725–733.

Puhan MA, Frey M, Büchi S, Schünemann HJ. (2008). The minimal important difference of the hospital anxiety and depression scale in patients with chronic obstructive pulmonary disease. *Health and Quality of Life Outcomes*, *6*, 1–6.

Strand LI, Ljunggren AE, Bogen B, Ask T, Johnsen TB. (2008). The Short-Form McGill Pain Questionnaire as an outcome measure: Test-retest reliability and responsiveness to change. *European Journal of Pain*, *12*(7), 917–925.

Grafton KV, Foster NE, Wright CC. (2005). Test-Retest Reliability of the Short-Form McGill Pain Questionnaire Assessment of Intraclass Correlation Coefficients and Limits of Agreement in Patients with Osteoarthritis. *Clin J Pain*, *21*, 73–82.

Tsai A. Hur, S A, Wong A, et al. (2021). Minimum important difference of the EQ-5D-5L and EQ-VAS in fibrotic interstitial lung disease. *Thorax*, *76*(1), 37–43.

Wolfe F, Michaud K. (2007). Assessment of Pain in Rheumatoid Arthritis: Minimal Clinically Significant Difference, Predictors, and the Effect of Anti-Tumor Necrosis Factor Therapy. *The Journal of Rheumatology*, *34*(8), 1674–1683.

Supplementary Figure 1. PK analysis RCT and EXT

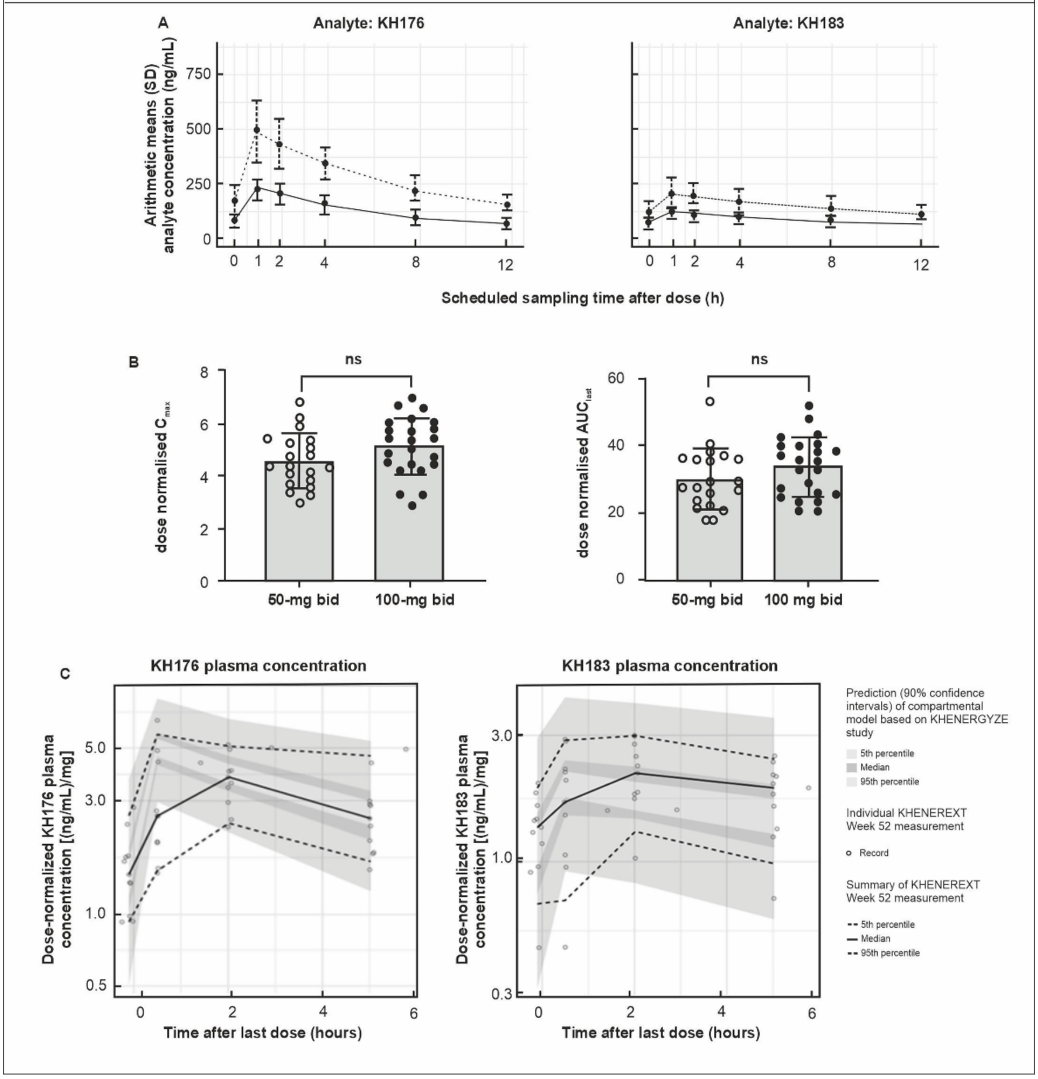

Panel A; Arithmetic mean (SD) concentration profiles of KH176 (parent compound) and KH183 of the RCT arm. Solid line; 50 mg dose, dotted line; 100 mg dose

Panel B; No significant differences in dose normalised Cmax (left) and exposure (AUClast (right)) between the 50 mg and 100 mg bid dosing in the RCT arm.

Panel C; Comparison of simulations based on the RCT arm and observations from the EXT arm for KH176 and KH183 plasma concentration. Data is plotted as dose normalised concentrations.

Supplementary Table 1 Cut-off values for the affected range and MCID values

Outcome measure	Subscale	Affected range	MCID	Reference Footnote
**Cogstate**		<-0.5	>0.5	[1]
**BDI**		>10	>17.5% of PD value	(Button et al., 2015)
**CFQ**		>43	>0.5 SD of PD value	(Broadbent et al., 1982)
**NQF**		>50	>5	[2] (Nowinski et al., 2016)
**NMDAS**		>10	All improvement is regarded as clinically important	(De Laat et al., 2021)
**MPQ**		All pain is regarded as affected	>5.2	(Grafton et al., 2005; Hawker et al., 2011; Strand et al., 2008; Wolfe & Michaud, 2007)
**SF-12**	PCS	<50	>5	[2]
	MCS	<50	>5	
**RAND SF36**	PF	<70,6	>5	(Angst et al., 2001)
	VT	<52,2	>5	
	SF	<78,8	>5	
	RP	<53	>5	
	ER	<65,8	>5	
	MH	<70,4	>5	
	BP	<70,8	>5	
	GH	<57	>5	
	HC	all	All improvement is regarded as clinically important	
**EQ-5D-5L**[3]	VAS	<82,4	>9,7	(Huber et al., 2016; Janssen et al., 2019; Jensen et al., 2023; Tsai et al., 2021)
	utility	[3]	-	
**HADS-A**	Anxiety	>7	>1,32	(Herrmann, 1997; Puhan et al., 2008)
**HADS-D**	Depression	>7	>1,4	
**5xSST**		>9.1 sec	> -2.3 sec	(Meretta et al., 2007, Klukowska et al, 2021)[4]
**Mini-BESTest**		<24,8	>3	(Dahl & Thorburn, 2001)
**Hand Strength**		Male: <42.8 (right) <40.9 (left) Female: <25.3 (right) <24.0 (left)	>5	
**UPSIT**		Male: <34 Female: <35	-	
**TAP**		-	-	

BDI=beck depression inventory; BP=bodily pain; CFQ=cognitive failure questionnaire; EQ-5D-5L=EuroQol (quality of life) 5-Dimensions-5 levels; ER=emotional role; GH=general health; HADS-A=hospital anxiety and depression scale-anxiety subscale; HADS-D=hospital anxiety and depression scale-depression subscale; HC=health change; MCID=minimal clinically important difference; MCS=mental component score; MH=mental health; MPQ=McGill Pain Questionnaire; NFQ=Neuro-QoL(quality of life) SF-Fatigue; NMDAS=newcastle mitochondrial disease adults scale; PCS=physical component score; PD=predose; PF=physical functioning; RP=role physical; SF=social functioning; SD=standard deviation; SF-12=12-Item Short Form Survey; SF-36=36-Item Short Form Survey; SST=sit-to-stand test; TAP=test of attentional performance; UPSIT=university of pennsylvania smell identification test; VAS=visual analog scale; VT=vitality.

[1] Z-score: 0 = mean, -1=1 SD worse than mean
[2] T-score: 50 = mean, -10 = 1 SD worse than mean
[3] EQ-5D-5L utility, age-dependent norm-based score, no affected range defined
[4] Most conservative threshold known in the literature applied

Supplementary Table 2 Efficacy baseline classification for norm-based parameters (RCT)

Parameter	Baseline Category	ITT		
		Placebo bid<br>n=24 (%)	50-mg bid<br>n=26 (%)	100-mg bid<br>n=25 (%)
BDI Total Score	Affected	16 (69.6)	13 (50.0)	15 (60.0)
	Normal	7 (30.4)	13 (50.0)	10 (40.0)
CFQ Total score	Affected	11 (47.8)	13 (50.0)	13 (52.0)
	Normal	12 (52.2)	13 (50.0)	12 (48.0)
HADS-A Score	Affected	9 (37.5)	11 (42.3)	9 (36.0)
	Normal	15 (62.5)	15 (57.7)	16 (64.0)
HADS-D Score	Affected	10 (41.7)	8 (30.8)	10 (40.0)
	Normal	14 (58.3)	18 (69.2)	15 (60.0)
Neuro-QoL Total	Affected	16 (66.7)	16 (61.5)	17 (68.0)
	Normal	8 (33.3)	10 (38.5)	8 (32.0)
SF-12: MCS	Affected	17 (77.3)	18 (81.8)	19 (79.2)
	Normal	5 (22.7)	4 (18.2)	5 (20.8)
SF-12: PCS	Affected	19 (86.4)	18 (81.8)	18 (75.0)
	Normal	3 (13.6)	4 (18.2)	6 (25.0)
NMDAS: Total	Mild/Moderate	10 (41.7)	11 (42.3)	13 (52.0)
	Severe	14 (58.3)	15 (57.7)	12 (48.0)

BDI=beck depression inventory; Bid=twice daily; CFQ=cognitive failure questionnaire; HADS-A=hospital anxiety and depression scale-anxiety subscale; HADS-D=hospital anxiety and depression scale-depression subscale; ITT=intent-to-treat; MCS=mental component score; NMDAS=newcastle mitochondrial disease adults scale; PCS=physical component score; PP=per-protocol; QoL=quality of life; RCT=randomized control trial; SF-12=12-item short form survey.

Allocation to baseline categories was done based on the predose value of each treatment period.

Supplemental Table 3 Characteristics of patients who participated in the randomized controlled (RCT) and the extension (EXT) arm of the Phase 2B program (see also Figure 1 and Table 1).

			Symptoms													NMDAS (predose)		Heteroplasmy level			Study participation	
Subject	Phenotype	Gender	Diabetes	CPEO	Hearing loss	Exercise intolerance	Myopathy	PMW	Depression	Fatigue	Headache	Migraine	Hypertension	CNS	GI	RCT	EXT	Blood	UEC	Muscle	RCT	EXT
1	MIDD	F	x		x	x				x			x	x	x	22	25	9	63		x	x
2	MIDD	M	x		x								x		x	13		27			x	
3	MIDD	M	x		x	x				x	x		x		x	24	20	20	93		x	x
4	MIDD	F	x		x		x				x					14	25	34	60		x	x
5	MELAS	F				x				x		x		x	x	17	13	24	49		x	x
6	MIDD	F	x		x	x				x					x	16	22	30	75		x	x
7	MIDD	M	x		x					x					x	27	22		93		x	x
8	MIXED	F				x						x			x	23		48	81		x	
9	MIDD	F	x		x	x	x				x		x			21	12	27	67		x	x
10	MIDD	M	x	x	x	x		x	x	x						16	28	34	82		x	
11	MIXED	F	x		x	x	x		x	x	x	x		x	x	54	37	22	59		x	
12	MIDD	F	x		x						x	x			x	18		62	74		x	
13	MIDD	F	x		x						x				x	24		8	52		x	
14	MIXED	F								x						25		*			x	
15	MIXED	M			x		x								x	42		14	77		x	
16	MIDD	F	x		x							x			x	29	31	35	56		x	
17	MIXED	F			x				x			x			x	46		21	74		x	
18	MIDD	F	x		x								x			13	15	**	**		x	x
19	MIDD	M	x		x								x	x	x	33	27	25			x	x
20	MIXED	F			x			x			x	x				14		20	80		x	
21	MIXED	F		x	x	x						x		x		32	41		70		x	x
22	MIXED	F						x			x					15	15	20	30		x	
23	MIXED	M	x			x										21	21	15	75		x	x
24	MIXED	F			x	x			x	x	x			x		17		20	35		x	
25	CPEO	F		x	x	x					x	x	x			14				75	x	
26	MIDD	F	x		x	x		x	x			x		x		18		38	62		x	
27	MIDD	F	x		x	x				x					x	15	14	12			x	x
	PERCENTAGE		63%	11%	81%	52%	15%	15%	19%	37%	37%	37%	26%	26%	52%							

CNS=central nervous system; CPEO=chronic progressive external ophthalmoplegia; EXT= open-label extension study; GI=gastrointestinal; MELAS=mitochondrial encephalopathy, lactic acidosis, and stroke-like episodes; MIDD= maternally inherited diabetes mellitus, and deafness; NMDAS=newcastle mitochondrial disease adults scale; PMW= Proximal muscle weakness; RCT=randomized controlled trial; UEC=urinary epithelial cells.

* confirmed by TaqMan genotyping; * *confirmed by Sanger sequencing.

Supplementary Table 4 Summary statistics of key PK parameters by analyte and dose level of the RCT

Group	Analyte	n	T_{max} [h][1]	C_{max} [ng/mL][2]	AUC_{last} [ng/mL*h][2]
50-mg	KH176	23	1.18 (33.00)	218.8 (21.61)	1458.3 (27.5)
	KH183	23	1.18 (41.74)	116.8 (38.96)	999.5 (40.3)
100-mg	KH176	23	1.27 (53.81)	493.4 (24.37)	3257.7 (27.4)
	KH183	23	1.4 (51.15)	197.4 (41.23)	1699 (36.5)

AUC_{last}=area under maximal concentration-time curve until the last time with a concentration above the limit of quantification; C_{max}= maximum concentration; PK=pharmacokinetic; RCT=randomized control trial; T_{max}=Time of C_{max}.

[1]Arithmetic summary statistics indicated as mean (CV%).
[2]Geometric summary statistics indicated as mean (CV%).

Supplemental Table 5 Change from baseline and change from placebo for RCT

				ITT		
				**Placebo**	**50-mg bid**	**100-mg bid**
**Cogstate IDN**	**Baseline**		mean (sd)	2.7716 (0.1024)	2.7682 (0.0731)	2.7742 (0.0834)
	**Mixed Model analysis**		CFB (*p* value)	-0.134 (0.266)	0.169 (0.150)	-0.103 (0.374)
			95% CI	-0.376, 0.109	-0.066, 0.405	-0.338, 0.132
			CFP (*p* value)		0.303 (0.073)	0.030 (0.853)
			95% CI		-0.030, 0.636	-0.304, 0.365
	**Modified Mixed Model Analysis**	More affected	CFB (*p* value)	0.015 (0.931)	0.494 (**0.004**)	-0.129 (0.408)
			95% CI	-0.333 - 0.362	0.174 - 0.813	-0.445 - 0.187
			CFP (*p* value)		0.479 (**0.034**)	-0.143 (0.499)
			95% CI		0.040 - 0.918	-0.576 - 0.288
		Less affected	CFB (*p* value)	-0.293 (0.072)	-0.187 (0.243)	-0.015 (0.930)
			95% CI	-0.616 - 0.029	-0.510 - 0.136	-0.350 - 0.321
			CFP (*p* value)		0.106 (0.608)	0.279 (0.198)
			95% CI		-0.316 - 0.528	-0.156 - 0.714
**Cogstate global composite**	**Baseline**		mean (sd)	-0.566 (0.774)	-0.509 (0.643)	-0.473 (0.781)
	**Mixed Model analysis**		CFB (*p* value)	0.155 (**0.024**)	0.107 (0.088)	-0.043 (0.502)
			95% CI	0.022, 0.288	-0.017, 0.232	-0.173, 0.087
			CFP (*p* value)		-0.048 (0.576)	-0.198 **(0.035)**
			95% CI		-0.221, 0.126	-0.380,-0.015
	**Modified Mixed Model Analysis**	More affected	CFB (*p* value)	0.2434 **(0.0048)**	0.1116 (0.2165)	0.1362 (0.1313)
			95% CI	0.08144 , 0.4054	-0.06968, 0.2928	-0.04362 , 0.3161
			CFP (*p* value)		-0.1318 (0.2337)	-0.1072 (0.3079)
			95% CI		-0.3543 , 0.09062	-0.3192 , 0.1049
		Less affected	CFB (*p* value)	0.1021 (0.3251)	0.05626 (0.4795)	-0.2546 **(0.0081)**
			95% CI	-0.1073 , 0.3114	-0.1052 , 0.2177	-0.4367 , -0.07235
			CFP (*p* value)		-0.04580 (0.7176)	-0.3566 **(0.0127)**
			95% CI		-0.3037 , 0.2121	-0.6300 , -0.08319
**TAP (with alarm)**	**Baseline**		mean (sd)	329.0 (85.4)	341.0 (111.4)	329.2 (111.0)
	**Mixed Model analysis**		CFB (*p* value)	-18.3978 (0.2201)	-18.0998 (0.2097)	10.6007 (0.4645)
			95% CI	-48.5223 , 11.7267	-47.0494 , 10.8497	-18.7929 , 39.9942
			CFP (*p* value)		0.2980 (0.9863)	28.9985 (0.1056)
			95% CI		-34.9794 , 35.5754	-6.5757 , 64.5727
	**Modified Mixed Model Analysis**	More affected	CFB (*p* value)	-38.9705 **(0.0304)**	**-66.9582 (0.0008)**	-2.2662 (0.9063)
			95% CI	-73.9414 -3.9996	-103.01 , -30.9079	-41.5112 , 36.9787
			CFP (*p* value)		-27.9877 (0.1969)	36.7042 (0.1084)
			95% CI		-71.4641 , 15.4887	-8.6919 , 82.1003
		Less affected	CFB (*p* value)	10.2517 (0.6042)	23.1825 (0.1792)	22.1208 (0.1924)
			95% CI	-29.9641 50.4676	-11.3682 , 57.7331	-11.8858 , 56.1274

			CFP (*p* value)		12.9307 (0.5721)	11.8691 (0.5942)
			95% CI		-33.5890 , 59.4505	-33.4237 , 57.1618
**TAP (without alarm)**	**Baseline**		mean (sd)	339.4 (88.5)	353.1 (105.2)	344.9 (106.6)
	**Mixed Model analysis**		CFB (*p* value)	-10.8555 (0.4045)	-7.7724 (0.5390)	1.0216 (0.9352)
			95% CI	-37.2187 , 15.5078	-33.4690 , 17.9242	-24.6056 , 26.6487
			CFP (*p* value)		3.0830 (0.8174)	11.8770 (0.3683)
			95% CI		-24.1367 , 30.3028	-14.8221 , 38.5762
	**Modified Mixed Model Analysis**	More affected	CFB (*p* value)	-28.9576 (0.0998)	-8.5689 (0.5990)	0.4841 (0.9778)
			95% CI	-63.8508 , 5.9356	-41.7074 , 24.5695	-34.9098 , 35.8781
			CFP (*p* value)		20.3887 (0.3056)	29.4417 (0.1486)
			95% CI		-19.7599 , 60.5373	-11.2443 , 70.1277
		Less affected	CFB (*p* value)	5.8964 (0.7338)	-5.9018 (0.7290)	4.1430 (0.7926)
			95% CI	-29.4137 , 41.2066	-40.5991 , 28.7955	-27.9608 , 36.2469
			CFP (*p* value)		-11.7982 (0.5656)	-1.7534 (0.9244)
			95% CI		-53.5288 , 29.9324	-39.4439 , 35.9370
**HADS-A**	**Baseline**		mean (sd)	6.46 (3.83)	6.69 (4.67)	6.52 (4.14)
	**Mixed Model analysis**		CFB (*p* value)	0.4172 (0.3627)	0.1361 (0.7528)	0.4344 (0.3273)
			95% CI	-0.5095 , 1.3438	-0.7439 , 1.0161	0.4610 , 1.3298
			CFP (*p* value)		-0.2811 (0.6116)	0.01722 (0.9757)
			95% CI		-1.4067 , 0.8445	-1.1372 , 1.1716
	**Modified Mixed Model Analysis**	Normal	CFB (*p* value)	0.6609 (0.3994)	-0.2511 (0.7060)	0.6085 (0.4318)
			95% CI	-0.9265 , 2.2482	-1.6065 , 1.1043	-0.9601 , 2.1771
			CFP (*p* value)		-0.9120 (0.3254)	-0.05237 (0.9589)
			95% CI		-2.7842 , 0.9602	-2.1234 , 2.0186
		Affected	CFB (*p* value)	0.2850 (0.6225)	0.4569 (0.4332)	0.2894 (0.5998)
			95% CI	-0.8927 , 1.4628	-0.7244 , 1.6382	-0.8324 , 1.4112
			CFP (p value)		0.1719 (0.8176)	0.004406 (0.9949)
			95% CI		-1.3472 , 1.6910	-1.4049 , 1.4137
**HADS-D**	**Baseline**		mean (sd)	6.58 (3.91)	6.35 (4.57)	6.64 (4.02)
	**Mixed Model analysis**		CFB (*p* value)	0.283 (0.583)	-0.332 (0.505)	0.091 (0.857)
			95% CI	-0.763 , 1.329	-1.344 , 0.679	-0.934 , 1.115
			CFP (*p* value)		-0.615 (0.370)	-0.192 (0.784)
			95% CI		-2.004 , 0.773	-1.621 , 1.236
	**Modified Mixed Model Analysis**	Normal	CFB (*p* value)	0.378 (0.635)	-2.343 **(0.013)**	-0.090 (0.908)
			95% CI	-1.243 , 1.998	-4.138 , -0.548	-1.684 , 1.503
			CFP (*p* value)		-2.721 **(0.026)**	-0.468 (0.681)
			95% CI		-5.081 , -0.361	-2.783 , 1.847
		Affected	CFB (*p* value)	0.113 (0.863)	0.638 (0.278)	-0.037 (0.953)
			95% CI	-1.225 , 1.450	-0.546 , 1.821	-1.330 , 1.256
			CFP (p value)		0.525 (0.546)	-0.150 (0.864)
			95% CI		-1.241 , 2.291	-1.933 , 1.633
**Beck Depression Inventory (BDI)**	**Baseline**		mean (sd)	15.75 (10.41)	14.31 (10.34)	15.40 (9.34)
			CFB (*p* value)	0.391 (0.708)	-1.010 (0.315)	-1.969 (0.055)

	**Mixed Model analysis**		95% CI	-1.735 , 2.517	-3.040 , 1.020	-3.982 , 0.043
			CFP (*p* value)		-1.401 (0.288)	-2.360 (0.071)
			95% CI		-4.058 , 1.255	-4.940 , 0.219
	**Modified Mixed Model Analysis**	Affected	CFB (*p* value)	0.297 (0.811)	-2.557 (0.083)	-3.651 (**0.007**)
			95% CI	-2.228 , 2.822	-5.476 , 0.362	-6.229 , -1.073
			CFP (*p* value)		-2.854 (0.110)	-3.948 (**0.014**)
			95% CI		-6.399 , 0.690	-7.038 , -0.859
		Normal	CFB (*p* value)	0.945 (0.609)	0.696 (0.614)	-0.090 (0.953)
			95% CI	-2.808 , 4.697	-2.111 , 3.503	-3.202 , 3.022
			CFP (*p* value)		-0.249 (0.905)	-1.035 (0.633)
			95% CI		-4.489 , 3.992	-5.446 , 3.377
**Neuro-QoL Fatigue SF**	**Baseline**		mean (sd)	26.00 (7.08)	25.04 (6.73)	25.36 (6.94)
	**Mixed Model analysis**		CFB (p value)	-0.4203 (0.6775)	-1.7517 (**0.0830**)	-2.2450 (**0.0295**)
			95% CI	-2.4775 , 1.6368	-3.7498 , 0.2463	-4.2471 , -0.2429
			CFP (p value)		-1.3314 (0.2773)	-1.8247 (0.1340)
			95% CI		-3.8001 , 1.1374	-4.2513 , 0.6019
	**Modified Mixed Model Analysis**	Affected	CFB (p value)	-1.6231 (0.2042)	-2.5823 (0.0580)	-3.0203 (**0.0186**)
			95% CI	-4.1868 , 0.9407	-5.2591 , 0.09454	-5.4921 , -0.5486
			CFP (p value)		-0.9592 (0.5583)	-1.3973 (0.3463)
			95% CI		-4.2893 , 2.3709	-4.3954 , 1.6009
		Normal	CFB (p value)	1.8551 (0.3011)	-0.5074 (0.7419)	-0.4319 (0.8079)
			95% CI	-1.7632 , 5.4734	-3.6455 , 2.6308	-4.0514 , 3.1876
			CFP (p value)		-2.3625 (0.2687)	-2.2870 (0.3083)
			95% CI		-6.6640 , 1.9390	-6.8160 , 2.2420
**UPSIT**	**Baseline**		mean (sd)	28.83 (4.69)	29.31 (3.90)	29.04 (4.52)
	**Mixed Model analysis**		CFB (p value)	1.1974 (0.0628)	-0.1836 (0.7579)	0.2700 (0.6527)
			95% CI	-0.06909 , 2.4640	-1.3965 , 1.0294	-0.9508 , 1.4908
			CFP (p value)		-1.3810 (0.1259)	-0.9274 (0.2953)
			95% CI		-3.1773 , 0.4153	-2.7141 , 0.8593
	**Modified Mixed Model Analysis**	More affected	CFB (p value)	2.0277 (**0.0313**)	-0.1144 (0.8855)	0.8730 (0.3052)
			95% CI	0.1973 , 3.8581	-1.7330 , 1.5043	-0.8445 , 2.5905
			CFP (p value)		-2.1421 (0.0813)	-1.1547 (0.3562)
			95% CI		-4.5709 , 0.2868	-3.6844 , 1.3750
		Less affected	CFB (p value)	0.5919 (0.5036)	-0.2597 (0.7674)	-0.5088 (0.5566)
			95% CI	-1.2042 , 2.3881	-2.0482 , 1.5288	-2.2670 , 1.2494
			CFP (p value)		-0.8516 (0.5030)	-1.1007 (0.3811)
			95% CI		-3.4324 , 1.7292	-3.6432 , 1.4419
**Cognitive Failure Questionnaire (CFQ)**	**Baseline**		mean (sd)	40.48 (20.50)	43.73 (22.30)	45.08 (20.30)
	**Mixed Model analysis**		CFB (*p* value)	1.033 (0.530)	-2.572 (0.104)	-4.513 (**0.007**)
			95% CI	-2.304 , 4.370	-5.715 , 0.570	-7.700 , -1.326
			CFP (*p* value)		-3.605 (0.100)	-5.546 (**0.015**)
			95% CI		-7.948 , 0.738	-9.893 , -1.199
			CFB (*p* value)	1.840 (0.450)	**-6.448 (0.005)**	-3.927 (0.082)

	**Modified Mixed Model Analysis**	More affected	95% CI	-3.104 , 6.785	-10.822 , -2.074	-8.388 , 0.533
			CFP (*p* value)		-8.288 (**0.011**)	-5.768 (0.077)
			95% CI		-14.530 , -2.046	-12.209 , 0.673
		Less affected	CFB (*p* value)	0.445 (0.844)	0.159 (0.942)	-4.039 (0.080)
			95% CI	-4.162 , 5.052	-4.311 , 4.629	-8.606 , 0.528
			CFP (*p* value)		-0.286 (0.923)	-4.484 (0.142)
			95% CI		-6.342 , 5.769	-10.568 , 1.600
**Headache***	Number of headache days		mean (sd)	6.4 (6.6)	5.5 (5.5)	4.9 (5.2)
			95% CI	3.6 , 9.2	3.3 , 7.7	2.8 , 7.1
			CFP (sd)		-0.7 (3.8)	-0.8 (3.5)
			95% CI		-2.3 , 0.9	-2.3 , 0.7
	Headache duration (h)		mean (sd)	6.59 (5.53)	5.92 (3.01)	4.62 (2.41)
			95% CI	3.83 , 9.34	4.47 , 7.37	3.38 , 5.86
			CFP (sd)		-0.86 (6.36)	-0.57 (2.81)
			95% CI		-4.13 , 2.41	-2.13 , 0.99
	Headache intensity		mean (sd)	4.49 (1.74)	4.58 (1.53)	4.18 (1.71)
			95% CI	3.62 , 5.35	3.84 , 5.32	3.30 , 5.05
			CFP (sd)		0.11 (1.19)	-0.27 (0.72)
			95% CI		-0.50 , 0.72	-0.67 , 0.13
	Max intensity		mean (sd)	6.4 (2.1)	6.5 (2.1)	6.0 (2.1)
			95% CI	5.4 , 7.4	5.5 , 7.5	4.9 , 7.1
			CFP (sd)		0.1 (1.5)	-0.2 (1.0)
			95% CI		-0.7 , 0.9	-0.8 , 0.4
	Times medication used		mean (sd)	4.3 (5.1)	4.3 (4.2)	4.2 (4.5)
			95% CI	1.8 , 6.8	2.3 , 6.3	1.9 , 6.5
			CFP (sd)		0.4 (3.4)	-0.3 (3.0)
			95% CI		-1.4 , 2.1	-1.9 , 1.4
**SF12 MCS**	**Baseline**		mean (sd)	42.37 (8.40)	43.11 (8.60)	43.19 (7.10)
	**Mixed Model analysis**		CFB (p value)	0.954 (0.410)	-0.613 (0.599)	1.767 (0.124)
			95% CI	-1.392, 3.301	-2.984, 1.758	-0.522, 4.057
			CFP (p value)		-1.567 (0.345)	0.813 (0.627)
			95% CI		-4.919, 1.785	-2.594, 4.220
	**Modified Mixed Model Analysis**	Affected	CFB (p value)	2.3324 (0.0703)	0.3364 (0.7890)	1.2389 (0.3148)
			95% CI	-0.2079 , 4.8726	-2.2247 , 2.8976	-1.2482, 3.7260
			CFP (p value)		-1.9959 (0.2680)	-1.0935 (0.5577)
			95% CI		-5.6244 , 1.6326	-4.8839 , 2.6969
		Normal	CFB (p value)	-3.6745 (0.1206)	-3.3579 (0.1771)	1.4801 (0.5072)
			95% CI	-8.3840 , 1.0349	-8.3371 , 1.6213	-3.0502 , 6.0103
			CFP (p value)		0.3166 (0.9277)	5.1546 (0.1299)
			95% CI		-6.8004 , 7.4336	-1.6242 , 11.9334
**SF12 PCS**	**Baseline**		mean (sd)	38.49 (10.47)	38.92 (10.30)	40.50 (10.89)
			CFP (p value)	1.435 (0.109)	2.301 (**0.013**)	-0.768 (0.369)
			95% CI	-0.341, 3.211	0.535, 4.067	-2.497, 0.961

**Mixed Model analysis**		CFP (p value)<br>95% CI		0.866 (0.387)<br>-1.159, 2.892	-2.203 **(0.034)**<br>-4.227, -0.179
**Modified Mixed Model Analysis**	Affected	CFB (p value)	1.1964 (0.1995)	2.6126 **(0.0103)**	-0.5984 (0.5363)
		95% CI	-0.6734 , 3.0663	0.6742 , 4.5509	-2.5640 , 1.3672
		CFP (p value)		1.4161 (0.2011)	-1.7949 (0.1115)
		95% CI		-0.8050 , 3.6372	-4.0356 , 0.4459
	Normal	CFB (p value)	4.2085 (0.1298)	0.9744 (0.5982)	-0.8094 (0.6142)
		95% CI	-1.3251 , 9.7421	-2.7856 , 4.7344	-4.0752 , 2.4565
		CFP (p value)		-3.2341 (0.3264)	-5.0179 (0.0916)
		95% CI		-9.8882 , 3.4200	-10.9088 , 0.8730

Bid=twice daily; CFB=change from baseline; CFP=change from placebo; CI=confidential interval; HADS-A=hospital anxiety and depression scale - anxiety subscale; HADS-D=hospital anxiety and depression scale - depression subscale; IDN=identification task test; ITT=intent-to-treat; MCS= mental component score; PCS= physical component score; QoL= quality of life; RCT=randomized controlled trial; SF=short form; SF-12= 12-Item Short Form Survey; SD=standard deviation; TAP= Test of attentional performance; UPSIT= University of Pennsylvania Smell Identification Test.

For IDN data from an extreme outlier was excluded. Data of Hearing could not be presented in this manner because of data complexity. Headache data is presented without statistical analysis due to data complexity. NMDAS was not considered in this short duration study. P-values below 0.05 are indicated in bold.
Indicated with grey shading is the analysis according to the statistical analysis plan.

Supplemental Table 6 Primary and secondary outcomes results in the EXT

		FAS	Affected at PD	PD value	CFB 52 weeks							RM 52 weeks				total responders	responders > MCID
		n	n	Mean	SD	n	Mean	SD	95%CI		t-test	treatment estimate	95%CI		ANCOVA	n	n
Cogstate	IDN	12	8	-1.048	1.019	11	0.277	0.555	-0.096	0.650	0.129	0.246	-0.202	0.694	0.275	7	3
Cogstate	GMLT	12	1	0.512	0.517	10	0.121	0.636	-0.334	0.576	0.562	0.109	-0.143	0.362	0.386	6	3
Cogstate	ONB-speed	12	5	-0.433	1.264	11	0.238	0.476	-0.082	0.558	0.128	0.226	-0.087	0.538	0.152	8	2
Cogstate	ONB-accuracy	12	4	0.058	1.257	11	0.222	1.385	-0.709	1.152	0.607	0.286	-0.510	1.082	0.472	5	5
Cogstate	DET	12	7	-0.988	0.805	11	-0.070	1.158	-0.848	0.708	0.846	-0.064	-0.590	0.463	0.809	5	2
Cogstate	OCL	12	1	0.910	0.899	11	0.227	0.963	-0.420	0.873	0.453	0.229	-0.317	0.774	0.402	5	3
Cogstate	ISLT	12	1	0.551	0.816	10	-0.358	0.626	-0.806	0.089	0.104	-0.354	-0.779	0.072	0.101	3	0
Cogstate	Attention composite 1 (IDN+DET)	12	6	-1.018	0.873	11	0.103	0.796	-0.431	0.638	0.675	0.095	-0.312	0.502	0.639	6	3
Cogstate	Attention composite 2 (IDN+DET+ONB)	12	6	-0.823	0.978	11	0.148	0.626	-0.272	0.569	0.450	0.110	-0.231	0.451	0.516	6	2
Cogstate	Executive function composite	12	2	0.039	0.681	10	0.183	0.399	-0.103	0.468	0.182	0.187	-0.029	0.404	0.088	7	1
Cogstate	Learning/Working memory composite	12	1	0.484	0.895	11	0.224	0.879	-0.367	0.815	0.418	0.252	-0.299	0.803	0.361	6	5
Cogstate	Global Composite	12	3	-0.083	0.625	11	0.035	0.482	-0.320	0.327	0.981	0.018	-0.214	0.249	0.879	5	1
TAP	With alarm	12	-	322.60	69.700	11	-56.60	59.50	-96.60	-16.70	**0.010**	-53.00	-72.60	-33.40	**<0.0001**	10	-
TAP	Without alarm	12	-	312.30	55.00	11	-34.20	31.30	-55.20	-13.10	**0.005**	-32.00	-52.20	-11.80	**0.003**	9	-
HADS	Anxiety	12	2	5.100	2.80	11	-0.900	4.100	-3.700	1.900	0.480	-0.800	-2.700	-1.000	0.381	8	6
HADS	Depression	12	3	4.300	4.00	11	-0.700	3.800	-3.300	1.800	0.542	-0.600	-2.400	1.300	0.543	6	4
BDI	Affective	12	-	1.600	1.70	11	-0.500	1.800	-1.700	0.600	0.326	-0.500	-1.500	0.500	0.284	-	-
BDI	Cognitive	12	-	2.100	2.10	11	-0.700	1.300	-1.600	0.200	0.104	-0.700	-1.800	0.400	0.202	-	-

BDI	Somatic	12	-	7.100	4.40	11	-3.200	4.000	-5.900	-0.500	**0.026**	-2.900	-4.900	-1.000	**0.004**	-	-
BDI		12	7	10.800	7.60	11	-4.500	6.800	-9.100	0.100	0.056	-4.200	-7.500	-0.800	**0.016**	10	9
NMDAS	Section I	12		8.400	3.80	11	-1.400	2.300	-2.900	0.200	0.077	-1.400	-2.700	0.000	**0.043**	-	-
NMDAS	Section II	12		8.800	2.100	11	-0.800	1.300	-1.700	0.000	0.055	-0.900	-1.700	-0.100	**0.032**	-	-
NMDAS	Section III	12		4.200	4.300	11	0.600	1.400	-0.300	1.600	0.152	-2.000	0.000	1.500	0.154	-	-
NMDAS		12	12	21.400	8.000	11	-1.500	3.100	-3.600	0.500	0.130	-1.600	-3.600	0.400	0.118	6	6
SF-12	PCS	12	11	37.900	9.000	11	7.700	5.400	4.100	11.400	**0.001**	7.800	5.400	10.200	<0.0001	10	8
SF-12	MCS	12		47.100	5.800	11	-0.600	8.000	-6.000	4.700	0.799	-1.000	-5.400	3.500	0.666	6	2
SF36	Energy/fatigue	12	9	39.200	15.800	11	18.600	23.500	2.900	34.400	**0.025**	17.300	6.200	28.500	**0.003**	7	6
SF36	Emotional well-being	12	4	73.700	10.000	11	5.800	12.600	-2.600	14.300	0.156	5.800	-2.100	13.800	0.147	7	6
SF36	General health	12	11	39.200	14.400	10	7.000	8.600	0.900	13.100	**0.030**	6.900	0.800	13.000	**0.028**	6	5
SF36	Health change	12	-	47.900	19.800	11	27.300	30.500	6.800	47.800	**0.014**	25.000	8.800	41.100	**0.003**	6	6
SF36	Pain	12	10	55.600	20.200	11	18.900	20.000	5.500	32.300	**0.011**	18.000	7.400	28.600	**0.001**	8	8
SF36	Physical functioning	12	7	65.000	19.100	11	11.800	15.200	1.600	22.000	**0.028**	11.300	3.600	19.000	**0.005**	8	6
SF36	Role functioning/emotional	12	2	88.800	26.100	11	-6.100	32.700	-28.000	15.900	0.550	-9.100	-30.200	12.000	0.391	1	1
SF36	Role functioning/physical	12	11	18.800	28.500	11	38.600	42.400	10.200	67.100	**0.013**	36.400	13.700	59.200	**0.002**	7	7
SF36	Social functioning	12	11	57.500	17.900	10	21.100	21.300	5.900	36.300	**0.012**	20.900	10.100	31.700	**0.000**	7	7
NQF		12	7	52.500	5.400	11	-6.300	5.500	-10.000	-2.580	**0.004**	-6.000	-9.200	-2.800	**0.001**	9	5
UPSIT		12	7	29.000	2.100	11	0.700	2.600	-3.350	8.400	0.371	0.800	-2.200	3.700	0.596	8	-
CFQ		12	3	31.900	13.200	11	-4.600	7.400	-9.600	0.300	0.065	-3.900	-8.400	0.700	0.092	7	4
5xSST		11	11	13.750	3.020	11	-1.210	2.130	-2.640	0.220	0.088	-1.260	-2.400	-0.120	0.033	7	3
miniBest		10	6	25.900	2.100	9	2.400	1.400	1.300	3.500	**0.001**	2.400	1.000	3.800	**0.002**	8	3
handgrip strength	right	12	5	29.490	9.840	11	0.210	5.680	-2.780	2.510	0.911	0.140	-2.790	3.070	0.918	6	2
handgrip strength	left	12	6	26.800	8.870	11	1.460	3.620	0.340	4.970	**0.029**	2.840	0.240	5.440	0.034	10	4

MPQ	Sensory dimension	12	12	8.500	4.300	10	-4.500	4.400	-7.700	-1.300	**0.011**	-3.400	-6.600	-0.200	**0.036**	10	3
MPQ	Affective dimension	12	7	2.000	2.100	11	-0.600	2.600	-2.400	1.100	0.432	-0.100	-1.400	1.100	0.816	4	2
MPQ	PPI score	12	12	1.800	1.200	11	-0.900	1.400	-1.800	0.000	0.053	-0.900	-1.500	-0.200	**0.009**	6	-
MPQ	VAS	12	11	42.800	21.800	11	-21.500	13.300	-30.400	-12.500	**0.000**	-21.000	-32.100	-9.900	**0.000**	10	-
MPQ		12	12	10.500	5.800	10	-5.700	5.600	5.500	32.300	**0.011**	-3.700	-7.900	0.400	0.077	10	4
EQ-5D-5L	VAS	12	12	61.700	15.600	10	16.200	18.400	3.000	29.400	**0.021**	16.700	6.800	26.600	**0.002**	7	7
EQ-5D-5L	Index value	12	-	0.794	0.126	11	0.074	0.086	0.016	0.131	**0.017**	0.075	0.022	0.127	**0.007**	10	-

BDI=beck depression inventory; Bid=twice daily; CFB=change from baseline; CFQ=cognitive failure questionnaire; CI=confidential interval; DET=detection task; EXT=open-label extension study; FAS= full analysis set; GMLT=groton maze learning test; HADS-D=hospital anxiety and depression scale - depression subscale; IDN=identification task test; ISLT=international shopping list test; MCID=minimal clinically important difference; MCS=mental component score; NFQ=Neuro-QoL(quality of life) SF-Fatigue; UPSIT=university of pennsylvania smell identification test; NMDAS=newcastle mitochondrial disease adults scale; OCL=one card learning task; ONB=one back task; PCS=physical component score; PD=predose; PPI=present pain intensity; RM=repeated measures; SD=standard deviation; SF-12=12-item short form survey; SF-36=36-item short form survey; SST=sit-to-stand test; TAP=test of attentional performance; VAS=visual analog scale.

Supplemental Table 7 Most common TEAEs in RCT and EXT (safety analysis set)

	Placebo n=25 (%)	Sonlicromanol 50-mg bid n=27 (%)	Sonlicromanol 100-mg bid n=26 (%)	EXT n=27 (%)
Any TEAE	15 (60)	14 (52)	12 (46)	13 (87)
Mild	11 (44)	13 (48)	11 (42)	
Moderate	7 (28)	7 (26)	9 (35)	
Severe	0 (0)	0 (0)	0 (0)	
Most common TEAEs	In >1 patient in one of the 3 groups			In > 1 patient
COVID-19				8 (53)
Influenza				4 (27)
Upper respiratory tract infection	1 (4)	3 (11)	1 (4)	3 (20)
Muscle spasms				4 (27)
Medical device site reaction	3 (12)	3 (11)	3 (12)	
Vomiting	0 (0)	1 (4)	3 (12)	3 (20)
Abdominal pain upper	2 (8)	1 (4)	2 (8)	
Abdominal pain	2 (8)	0 (0)	0 (0)	2 (13)
Fatigue	2 (8)	1 (4)	2 (8)	
Pain in extremity	2 (8)	0 (0)	0 (0)	
Diarrhea	1 (4)	0 (0)	2 (8)	3 (20)
Palpitations	0 (0)	1 (4)	2 (8)	
Blood glucose fluctuations				3 (20)
Myalgia				3 (20)
Constipation				2 (13)
Nausea				2 (13)
Pyrexia				2 (13)
Dysaesthesia				2 (13)
Hyperglycemia				2 (13)

Bid,=twice daily; EXT= extension study; RCT=randomised controlled study; TEAE= treatment-emergent adverse events.
